# Preclinical safety and biodistribution of CRISPR targeting SIV in non-human primates

**DOI:** 10.1038/s41434-023-00410-4

**Published:** 2023-08-17

**Authors:** Tricia H. Burdo, Chen Chen, Rafal Kaminski, Ilker K. Sariyer, Pietro Mancuso, Martina Donadoni, Mandy D. Smith, Rahsan Sariyer, Maurizio Caocci, Shuren Liao, Hong Liu, Wenwen Huo, Huaqing Zhao, John Misamore, Mark G. Lewis, Vahan Simonyan, Elaine E. Thompson, Ethan Y. Xu, Thomas J. Cradick, Jennifer Gordon, Kamel Khalili

**Affiliations:** 1 Department of Microbiology, Immunology, and Inflammation, Center for NeuroVirology and Gene Editing, Lewis Katz School of Medicine, Temple University, Philadelphia, PA 19140 USA; 2 Excision BioTherapeutics, Inc., San Francisco, CA USA; 3 Center for Biostatistics and Epidemiology, Department of Biomedical Education and Data Science, Lewis Katz School of Medicine, Temple University, Philadelphia, PA 19140 USA; 4 BioQual, Inc., Rockville, MD USA; 5 Embleema, Metuchen, NJ USA

**Keywords:** Virology, Clinical genetics

## Abstract

In this study, we demonstrate the safety and utility of CRISPR-Cas9 gene editing technology for in vivo editing of proviral DNA in ART-treated, virally controlled simian immunodeficiency virus (SIV) infected rhesus macaques, an established model for HIV infection. EBT-001 is an AAV9-based vector delivering SaCas9 and dual guide RNAs designed to target multiple regions of the SIV genome: the viral LTRs, and the Gag gene. The results presented here demonstrate that a single IV inoculation of EBT-001 at each of 3 dose levels (1.4 × 10^12^, 1.4 × 10^13^ and 1.4 × 10^14^ genome copies/kg) resulted in broad and functional biodistribution of AAV9-EBT-001 to known tissue reservoirs of SIV. No off-target effects or abnormal pathology were observed, and animals returned to their normal body weight after receiving EBT-001. Importantly, the macaques that received the 2 highest doses of EBT-001 showed improved absolute lymphocyte counts as compared to antiretroviral-treated controls. Taken together, these results demonstrate safety, biodistribution, and in vivo proviral DNA editing following IV administration of EBT-001, supporting the further development of CRISPR-based gene editing as a potential therapeutic approach for HIV in humans.

## Introduction

Non-human primates (NHPs) have been used to understand lentiviral infection, pathobiology, pathogenesis and to predict clinical efficacy since the beginning of the HIV pandemic, and recently to evaluate eradication strategies and innovative approaches to cure HIV [1–3]. In the studies presented here, we employed simian immunodeficiency virus (SIV)-infected antiretroviral (ART)-treated Indian rhesus macaques to evaluate biodistribution, safety, and the ability of the CRISPR/Cas9 gene editing treatment (EBT-001) to target integrated SIV given in a single intravenous (IV) bolus infusion [4]. EBT-001 (encoding SIV targeting guide RNAs) is the simian homolog of EBT-101 (encoding HIV targeting guide RNAs) and is comprised of an adeno-associated virus serotype 9 (AAV9) encapsulated all-in-one CRISPR construct simultaneously expressing the SaCas9 endonuclease and dual guide RNAs (gRNAs) targeting the viral long terminal repeats (LTRs) and the Gag gene. EBT-101 is designed for the removal of large intervening integrated proviral SIV DNA sequences positioned between the intended CRISPR target sites, thereby generating 3 possible deletions: 5′LTR to Gag, Gag to 3′LTR, and 5′LTR to 3′LTR. The use of AAV9 delivery is supported by our initial studies in small and large animal models, in addition to a large body of evidence from previous gene therapy clinical trials where AAV9 has exhibited safety, efficacy and broad biodistribution properties [4–6]. Here, we present more comprehensive data on safety, including extensive off-target analyses, and biodistribution of multiple doses (10^12^, 10^13^, 10^14^ GC/kg) of EBT-001 and extended study (3 and 6 months) in an Indian rhesus macaque SIV model. This preclinical safety, biodistribution, and efficacy study demonstrated that a single IV administration of EBT-001 for SIV editing was well-tolerated and can broadly reach and excise viral reservoirs in NHPs without any observed off-target effects. These results support that a single IV inoculation of EBT-101 for HIV gene editing can be utilized to reach viral reservoirs in infected tissues throughout the body for the safe and specific in vivo editing of latent HIV proviral DNA.

## Materials and methods

### Study design

This study was approved by BIOQUAL’s IACUC, protocol number 18-036. Twelve male Indian rhesus macaque monkeys (Macaca mulatta) were obtained from the Caribbean Primate Research Center in Puerto Rico. Two studies were performed. The first was an *n* = 10 with animals randomized by a statistician into four treatment groups with 3 untreated, 3 treated with 1.4 × 10^12^ GC/kg EBT-001, and 4 treated with 1.4 × 10^13^ GC/kg EBT-001 (2 necropsied at 3 months and 2 at 6 months post EBT-001). Randomization was done using a permuted block randomization method with the restriction that social partner paired animals were in the same group. The second study was with an *n* = 2 using 1 log higher EBT-001 dose of 1.4 × 10^14^ GC/kg and no randomization was performed here. All animals were screened and were triple negative for Mamu-A*01, Mamu-B*08 and Mamu-B*17. All rhesus macaques were received at BIOQUAL’s Piccard Drive Facility, in Rockville, MD where all the in vivo studies described here were performed. The rhesus macaques were serology pre-screened for retroviruses (STLV and SIV), SA8 virus, SHF virus, and measles virus prior to the study. Animals were infected with SIVmac239 (a gift from Dr. Preston Marx, Tulane Primate Center). Animals were started on a daily ART regimen of tenofovir (TDF; #T018500; 15 mg/kg/animal), emtricitabine (FTC; #E525000; 50 mg/kg/animal), and dolutegravir (DTG; #D528800; 2.5 mg/kg/animal) (Toronto Research Chemicals) beginning on day 28 post infection and continued to necropsy. Animals were observed for morbidity, mortality, injury, and availability of food and water. Any animals in poor health were identified for further monitoring, clinical treatment, and possible euthanasia. Observations included, but were not limited to, evaluation of the skin, fur, eyes, ears, nose, oral cavity, thorax, abdomen, external genitalia, limbs and feet, respiratory and circulatory effects, autonomic effects such as salivation, nervous system effects including tremors, convulsions, reactivity to handling, neurological/behavioral effects, and unusual behavior. SIV viral load (limit of detection of 50 copies/mL) was measured in ethylenediaminetetraacetic acid (EDTA) plasma over the course of infection (Supplementary material and methods). Whole blood was collected on multiple days during the study period and submitted to IDEXX BioAnalytics, Inc. for complete blood count (CBC) analysis and for blood chemistries. Animals were dosed with EBT-001 at 6 months post SIV infection with a slow IV infusion at a rate of 1 mL/min. The amount of EBT-001 vector to be dosed was added to total of 100 mL of 1X phosphate-buffered saline (PBS) for each animal based on current body weight and dose (Table S2). Animals underwent a full necropsy at either 3 months or 6 months post-EBT-001 infusion (Supplementary material and methods). No blinding to animal groupings was done at BioQual.

### Bioinformatics, whole genome sequencing and analyses

This information can be found in the Supplementary material and methods (Supplemental material and methods and Figs. S1–S4).

### Biodistribution

A TaqMan-based qPCR assay was used to quantitate EBT-001 (vector) DNA in tissues (Charles River) and in blood over time (Supplementary material and methods).

### Excision assays

We used 2 nested PCR assays (5 G EXA and G3 EXA) to detect the different excision amplicons/products in internal organ and tissue samples obtained from macaques treated with EBT-001 (SIV AAV9 CRISPR/Cas9) gene therapy product (Supplementary material and methods). An estimate of the editing was calculated only for the 5 G products (Supplementary results, Tables S8 and S9, and Fig. S6). Research staff was blinded to animal groupings during excision assays and analyses.

### Cytokine analyses

EDTA plasma samples were analyzed using the Mesoscale Discovery (MSD) assay. Each well of the plate is a working electrode surface that absorbs the capture reagent. The Cytokine Panel 1 (NHP, MSD v-plex K15057D) and Proinflammatory Panel 1 (NHP, MSD kit #K15056D) were utilized in the study and specifically validated for NHPs. First, plates were washed 3 times and incubated with calibrators/samples for 2 h with shaking. Plates were then washed and incubated with the detection antibody cocktail for 2 h. After the last washing step, plates were read and data analyzed using the MSD software.

### Statistical analyses

Continuous measures were presented by scatter plots and summarized as mean ± standard effort of the mean. For monkey weights, Group 1 (untreated) was compared to Groups 2, 3, and 4 combined (treated). A linear mixed-effects model was used to compare percent change in weight from pre-EBT-001 over time between treated vs untreated groups. Statistical significance was based on a two-sided alpha level of less than 0.05. The variance was similar between groups and the data are normally distributed.

### Code availability for off-target assessment

Nomination of off-target editing sites was performed using proprietary COTANA pipeline that is based on an open source tool Cas-OFFinder v2.4.1 (https://github.com/snugel/cas-offinder/), which provides similar search results [7]. The output chromosomal sites were locally re-aligned and scored using nuclease-off-target tool v3.0.0 (https://github.com/eli88fine/nuclease-off-target) with penalty match score scoring matrix shown in Table S1. For WGS analysis, HIVE (https://hive.aws.biochemistry.gwu.edu/dna.cgi?cmd=main) was used [8, 9].

## Results

### Development of safe and effective viral specific gRNAs

CRISPR gRNA designer tools were used to identify gRNAs that target LTR and Gag with minimal chance of unintended effects for the previously described HIV-directed guides [6, 10]. Previous research demonstrated efficacy in targeting HIV in culture and using an infected humanized mouse model with guide RNAs targeting LTR-1 and GagD [6]. For this work in rhesus macaques, gRNAs targeting SIV were chosen to represent the same regions as the HIV guides for these biodistribution and safety studies.

**Fig. 1:**
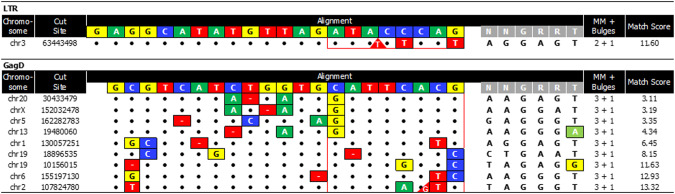
Multiple mismatches in sites identified from genome searches using LTR or Gag guide RNAs. All sites in the bioinformatic output from the rhesus macaque genome searches using SIV guide LTR allowing three differences and Gag allowing four differences are listed. The guide (colored) and PAM sequence (gray) are listed on top, the chromosomal sequence on the bottom with the locations and identity of mismatches shown in boxes. The two types of bulges are also displayed. Gaps in the chromosomal DNA relative the guide RNA are shown in red boxes with dashes. Additional nucleotides in the chromosomal DNA relative to the guide are shown in red triangles. The number of mismatches and bulges and match score for each chromosomal locations are listed in the right column. No loci were identified without mismatches in the proximal 8 nucleotides where differences are less tolerated, outlined in red. Moreover, none of the nominated off-target loci have a penalty match score less than 2 (right column). Bp base pair, chr chromosome, LTR long terminal repeats, MM mismatch, PAM protospacer-adjacent motif, SIV simian immunodeficiency virus.

The bioinformatics tool COTANA (CRISPR Off-Target Nomination & Analysis) exhaustively searched the rhesus macaque reference genome (NCBI genome assembly Mmul_10) for the most similar chromosomal sequences to the target site. COTANA output each nominated site with the specified number of differences between the gRNA and DNA sequence (Table 1). The output for the exhaustive search, including mismatches and possible bulges, contains few nominated sites in the rhesus macaque genome and very few with less than 4 differences (Supplementary materials and methods). The list of output sites also has few with low penalty match scores, which generally indicate a more possible chance of locating sequence-verified off-target sites. Penalty match scores were calculated based on the increasing number, type, and locations of the mismatches and bulges between the gRNA and chromosomal sequence (Fig. 1). When using gene editing to correct disease-causing mutations, one is generally limited to the closest few target sites. When targeting viral sequences for inactivation, there is much greater flexibility as multiple targeting strategies and series of target sites can be bioinformatically scanned to choose conserved viral target sites that have a much lower number and similarity of chromosomal locations [11–13]. Those gRNAs output many fewer sites than gRNAs targeting coding sequences, as seen in previous publications and when studying EBT-101 [11, 12].

**Table 1:** Few rhesus macaque chromosomal sites are output with increasing differences to the target sites.

Search settings:				
Differences	Mismatches	Bulges	LTR	GagD
Zero	0	0	–	–
One	1	0	–	–
"	0	1	–	–
Two	2	0	–	–
"	1	1	–	–
Three	3	0	–	–
"	2	1	1	–
Four	4	0	1	–
"	3	1	10	9
Five	5	0	39	24
"	4	1	159	144

The Mmul_10 rhesus macaque reference genome was searched for sequences with increasing number of differences (left column) to the 2 gRNA sequences (SIV LTR and SIV Gag) used in EBT-001 with COTANA. The table lists the number of rhesus macaque genomic sites output when searching with the LTR or Gag guides and saCas9 PAM (5′-NNGRRN-3′) using an increasing number of differences moving down in the table, including mismatches and bulges.

*COTANA* CRISPR Off-Target Nomination and Analysis, *gRNA* guide RNA, *LTR* long terminal repeat, PAM, protospacer-adjacent motif; saCas9, Staphylococcus aureus Cas9; SIV, simian immunodeficiency virus.

### Assessment of EBT-001 in NHP model of HIV

**Fig. 2:**
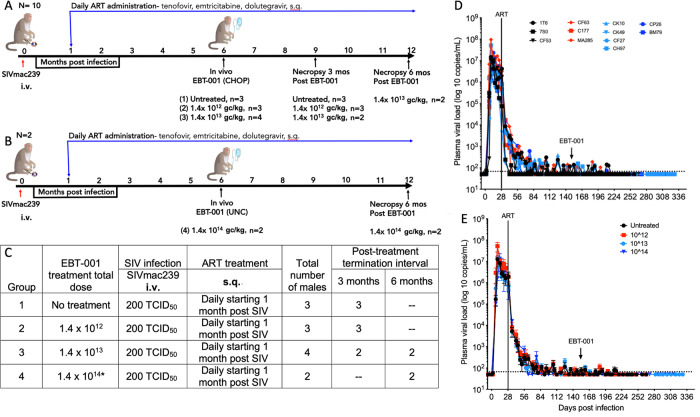
Non-human primate study design and viral loads. Initial study design *n* = 10 (**A**), follow-up study design *n* = 2 (**B**), study chart with groups 1-4 identified (**C**), Individual plasma viral loads (log10 copies/mL of plasma) (**D**) and mean and SEM of plasma viral loads of 4 Groups (**E**). Black is untreated Group 1; red is Group 2, 10^12^ GC/kg; light blue is Group 3, 10^13^ GC/kg; dark blue is Group 4, 10^14^ GC/kg. Mean and SEM are shown for the group at days post infection. ART anti-retroviral therapy, CHOP Children’s Hospital of Philadelphia, GC genome copies, IV intravenous, SIV simian immunodeficiency virus, sq subcutaneous, TCID_50_ median tissue culture infectious dose, UNC University of North Carolina.

**Fig. 3:**
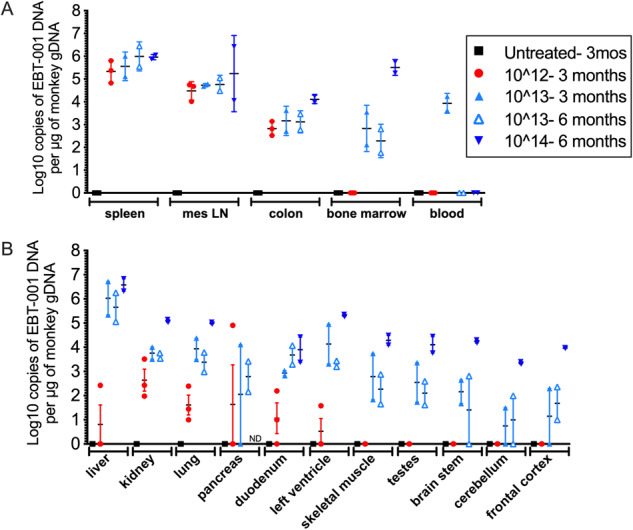
Biodistribution of EBT-001. EBT-001 vector DNA biodistribution as log10 copies of EBT-001 DNA per µg of monkey gDNA in major tissue reservoirs of HIV (**A**) and other tissues, including various brain regions (**B**). Symbols represent individual animals and mean and SEM are shown. gDNA, genomic DNA; SEM, standard error of the mean. Untreated 3 mos- untreated animals did not receive EBT-001 but where necropsied at the same time as the EBT-001 animals treated for 3 months. ND not determined.

In two matching studies, SIV-infected and antiretroviral-treated NHP were used as a large animal model to test AAV9-delivered, EBT-001’s ability to remove integrated SIV DNA. In study one, a total of 10 male rhesus macaques (Macaca mulatta) were divided into 3 groups: Group 1 had no EBT-001 treatment, Group 2 with 1.4 × 10^12^ genome copies (GC)/kg of EBT-001, and Group 3 with 1.4 × 10^13^ GC/kg of EBT-001 (Fig. 2A). In study two, a higher dose (1.4 × 10^14^ GC/kg) of EBT-001 was given to 2 animals in Group 4 (Fig. 2B**)**. Both studies examined NHPs infected with SIVmac239 (200 TCID_50_) via the i.v. route and treated with a triple ART regimen (tenofovir (TDF), emtricitabine (FTC), and DTG) via the s.q. route (Fig. 2C). This regimen was selected for its similarity to how HIV-infected humans are treated to control HIV infection. ART began 28 days post-infection and all 12 animals remained on ART for the entire course of the study. Once viral levels were reduced to minimum levels in the blood, animals were maintained on only ART (Group 1) or treated with one of 3 doses of EBT-001 delivered by a single IV infusion (Fig. 3C, Supplementary material and methods, Table S2). All monkeys received full doses of EBT-001. One monkey (CK49) in Group 3 experienced shortness of breath and hypoxia after administration of anesthesia that worsened after infusion of EBT-001. The infusion of EBT-001 was paused while the monkey was treated for acute pulmonary edema and recovered. The remaining dose of EBT-001 was delivered several days later without any complications. This was an adverse reaction due to the anesthetic agent, Dexdomitor because the re-challenge of EBT-001 was well-tolerated and the symptoms were consistent with adverse reactions to Dexdomitor. One animal in Group 2 developed a skin rash after EBT-001 infusion. Plasma viral RNA was measured throughout the course of infection and after EBT-001 (Fig. 2D, E).

Plasma samples were assayed for neutralizing antibodies to AAV9 prior to the study and at necropsy (Table S4). EBT-001 treatment was associated with increases in AAV9 neutralizing antibodies 3 or 6 months after treatment relative to pre-dose values. The pre-dose titers (serum reciprocal dilution values) were low (<5 and 80), except CF63, which had a pre-dose titer of 320. The values at necropsy ranged from 80-5120 in EBT-001 treated animals and were highest in the monkeys sacrificed at 6 months post-EBT-001. Of note, CF63’s titer did not change after EBT-001 treatment even though it was the highest at baseline.

All monkeys survived until the scheduled euthanasia (3 or 6 months post EBT-001) when a full necropsy was performed on all animals (Supplementary material and methods). Overall, the gross necropsy observations were within normal range, except for a single animal (1T6, Group 1) from the ART-only treated group, who was dehydrated and had a thin body condition (2/5 body composition score). All findings were consistent with SIV infection (Table S5). No EBT-001-related gross or microscopic pathology findings were noted in a comprehensive histopathological evaluation of major organs performed by Charles River Laboratories, LLC. General microscopic findings attributed to chronic SIV infection were observed within all treatment groups and included minimal to mild lymphoid hyperplasia of the germinal centers and mantle zone of the mesenteric lymph node, lymphoid hyperplasia of the secondary lymphoid follicles in the white pulp of the spleen, and mononuclear cell infiltration of the heart, kidney, and lung. Additionally, there was lymphoid hyperplasia of the periarteriolar lymphoid sheaths of the spleen in animal BM79 (Group 4). There were no apparent differences in the frequency or severity of these findings in monkeys treated with ART only or in monkeys treated with ART in combination with EBT-001. In a separate neuropathological evaluation performed by Experimental Pathology Laboratories, Inc., findings in all SIV-infected rhesus macaques were few and consisted primarily of focal minimal findings of microgliosis and less commonly astrocytosis and/or sparse perivascular mononuclear cell infiltrates. Overall, the microscopic findings were consistent with chronic SIV infection and were observed with similar frequency in control SIV-infected animals examined at the same timepoints from another EBT-001 study. The findings were therefore not attributed to treatment with EBT-001.

### Detection of vector DNA in tissues

To assess the biodistribution of the vector in tissues at necropsy, a TaqMan-based qPCR assay was used to quantitate EBT-001 (vector) DNA (Charles River) (Fig. 3A, B). All DNA biodistribution and vector shedding samples collected from the untreated animals (Group 1) were negative, demonstrating the contamination was well controlled from animal dosing, necropsy, DNA extraction, and qPCR analysis in this study. Tissues that are considered major HIV/SIV reservoirs, including spleen, lymph nodes, colon, bone marrow compartment, and blood were examined for vector biodistribution (Fig. 3A). Levels of vector DNA in spleen ranged from 4.82–6.44 (log10 copies of EBT-001 DNA/ug of monkey DNA). For the mesenteric lymph nodes, the value ranged from 4.01–6.42 and for colon 2.52–4.24 (log10 copies of EBT-001 DNA/ug of monkey DNA). In bone marrow, vector DNA was undetectable in Group 2, 1.75–3.55 (log10 copies of EBT-001 DNA/ug of monkey DNA) in Group 3, and 2 logs higher in Group 4 with average 5.5 (log10 copies of EBT-001 DNA/ug of monkey DNA). In blood, where cells turnover rapidly, vector DNA was only found in the 2 animals from Group 3 that were necropsied at 3 months post-EBT-001 and was absent in animals in the same group that were necropsied at 6 months and in the higher dosed animal also sacrificed at 6 months (Group 4) (Fig. 3A). However, when biodistribution was examined serially in blood starting at 2 weeks post-EBT-001, vector DNA was detected in Groups 2 and 3 and decreased over time (Figure S5). The highest concentration of EBT-001 DNA was detected in the liver samples from Groups 3 and 4 (Fig. 3B). For the brain and other tissues, Group 2 was low or undetectable and distribution was increased in Group 3 but interestingly, levels in Group 4 were in the range of 4 (log10 copies of EBT-001 DNA/ug of monkey DNA). No evidence of vector shedding in stool or urine was observed (not shown). The qPCR results confirm that EBT-001 was distributed widely to blood and all major organs and tissues tested in animals dosed with the vector. Biodistribution of EBT-001 (DNA levels) was dose dependent, and the vector levels in each tissue and blood decreased over time, although persisting 6 months after injection in many tissues.

### Editing of proviral DNA fragments from blood cells and various solid organs

**Fig. 4:**
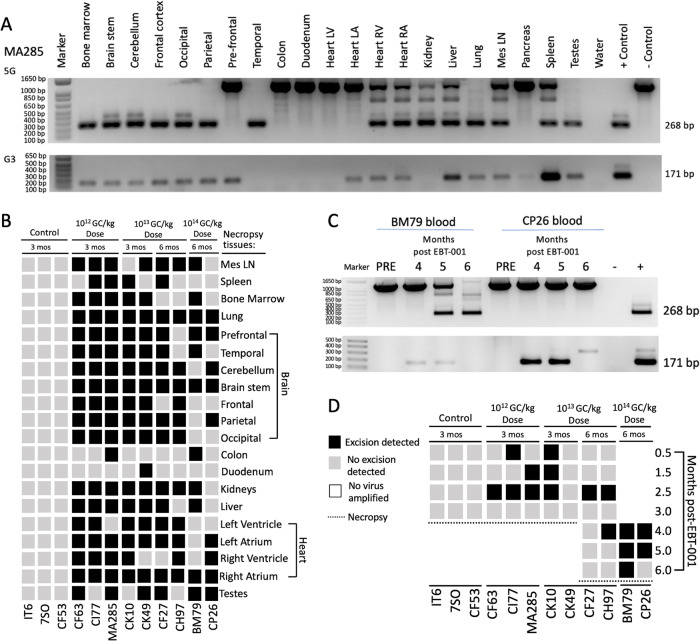
Proviral excision of SIV DNA in tissues and blood. Example of 5G and G3 PCR of necropsy tissues from monkey MA285 (**A**). Presence of viral excision (displayed as black boxes), absence of viral excision (gray boxes), or absence of full-length and excised product (white boxes) in multiple tissues at necropsy (**B**). Example of 5G and G3 PCR from blood of animals BM79 and CP26 pre-EBT-001 and 4, 5, and 6 months post-EBT-001 (**C**). Presence of viral excision (black box) or absence of viral excision (gray boxes) from the blood of animals after EBT-001 (**D**). Dotted line represents the necropsy timepoint of 3 months or 6 months. No blood was obtained from the 10^14^ GC/kg animals at 0.5-3.0 months post-EBT-001 due to COVID shutdown. GC genome counts, mes ln mesenteric lymph node, PCR polymerase chain reaction, SIV simian immunodeficiency virus.

SIV excision activity was assayed across treatment groups and tissues. The 2 gRNAs in EBT-001 target 3 locations in SIV and therefore excise 3 large intervening integrated proviral SIV segments. The LTR gRNA cuts both the 5′ and 3′LTR. As this guide is co-delivered along with the Gag gRNA, there is the possible removal of the intervening integrated proviral SIV DNA sequence between the 5′LTR to Gag, Gag to the 3′LTR and between the 5′LTR and 3′LTR. Two assays were used to demonstrate 5′LTR to Gag (5G) and Gag to 3′LTR (G3) excision in tissues and blood (see Supplementary material and methods and Supplementary Table 3). Evidence of excision was detected by the observation of nested PCR products of distinct DNA fragments of 268 or 171 bp resulting from the removal of intervening DNA sequences between 5′LTR to Gag (5G) or Gag to 3′LTR (G3), respectively (Fig. 4A, C). The 5 G assay also amplified the full-length SIV at an amplicon size of 1282 bp. Due to extensive homology between the 5′ and 3′LTR sequences and the lack of standardized flanking sequences due to the random chromosomal integration of SIV proviral DNA, it was not possible to measure the third excision product which removes the entire 5′LTR to 3′LTR portion. Both PCR-based excision assays (5G and G3) were completed (in duplicate by 2 operators) for blood and small sections of tissues and organs, including: bone marrow, brain (brain stem, cerebellum, frontal, occipital, parietal, prefrontal, temporal cortices), colon, duodenum, heart (left ventricle, left atrium, right ventricle, right atrium), kidneys, liver, lungs, mesenteric lymph nodes, spleen, and testes (Fig. 4, Tables S6, and S7). The 5G and G3 data are summarized in Table S6 for tissues and Table S7 for blood. In Fig. 4B for tissues and Fig. 4D for serial blood draws, positive excision, defined by the detection of the nested PCR product of distinct DNA fragments of 268 bp (5G) or 171 bp (G3) or both, is represented by a black box. No excision, defined by no detection of either of the nested PCR products of 268 bp (5G) or 171 bp (G3), is represented by a gray box. If no full-length (top band of 1282 bp in the 5G only) or excised product could be amplified (in both 5G and G3), then this is represented by a white box. This analysis demonstrated SIV DNA cleavage in a broad range of tissues, 3 and 6 months after vector treatment. SIV viral excision, 5′LTR to Gag or Gag to 3′LTR, was observed in some tissue for all EBT-001 treated animals. There was individual animal variability observed, as well as differences in excision in tissues. Excision was detected at 3 months and in some tissues at 6 months. No virus was detected in some tissue samples at the highest dose of EBT-001 (Group 4) at 6 months post-EBT-001, which may indicate the elimination of viral DNA from those sections of tissues. The results of the excision activity analysis showed SIV DNA cleavage in a broad range of tissues 3 and 6 months after vector treatment, and in blood 2–10 weeks after vector treatment, including the presence of SIV excision in the blood of one animal (BM79) at necropsy (Fig. 4D). Since the 5G EXA assay amplifies both the full-length virus (1282 bp) and the amplicon (268 bp) of the excision product after the removal of intervening DNA sequences between 5′LTR to Gag, we calculated the percent efficiency of the 5G excision (Tables S8 and S9, Fig. S6, Supplementary material and methods). We understand the limitations of this PCR-based assay, as it is semi-quantitative, and it is possible that the smaller excision products may be amplified more efficiently than the intact virus. In addition, this assay reflects only one of 3 possible excision products that can occur through EBT-001 cutting. Despite the limitations of assay detection, the results show varying excision abilities with several animals reported to have detectable excision in tissue sections examined, even at the lower doses.

### Weights, lymphocyte and monocyte counts, blood chemistries and cytokine analyses

**Fig. 5:**
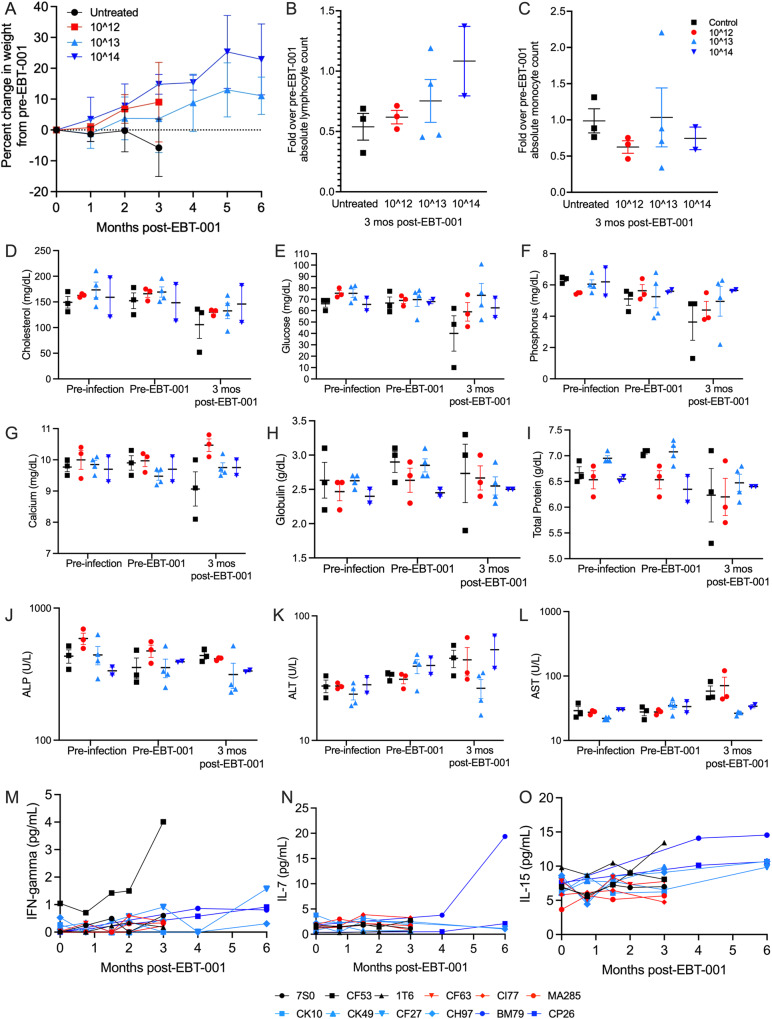
Clinical parameters of SIV-infected ART-treated animals untreated or treated with EBT-001. The mean (SEM) of animal’s weights are shown as percent change of pre-EBT-001 weight. Weights from EBT-001 treated (red, light blue, and dark blue lines) are significantly increased compared to untreated (black) (*P* = 0.029). A linear mixed-effects model was used to compare percent change in weight from pre-EBT-001 over time between treated vs untreated groups. Statistical significance was based on a 2-sided alpha level of less than 0.05 (**A**) The fold change in lymphocyte count from the CBC data over the average pre-EBT-001 values are shown. Each symbol is one animal and shown as the mean and SEM (**B**). The fold change in monocyte count from the CBC data over the average pre-EBT-001 values are shown. Each symbol is one animal and shown as the mean and SEM (**C**). The scatter dot plots show the mean and SEM, where each symbol represents an animal from pre-infection, pre-EBT-001 and 3 months post-EBT-001 for cholesterol (**D**), glucose (**E**), phosphorus (**F**), calcium (**G**), globin (**H**), total protein (**I**), ALP (**J**), ALT (**K**), and AST (**L**). Cytokines measured by MSD over time post-EBT-001 are shown for individual animals for plasma IFN-gamma (**M**), plasma IL-7 (**N**), plasma IL-15 (**O**). ALP alkaline phosphatase, ALT alanine transaminase, ART anti-retroviral therapy, AST aspartate transaminase, CBC complete blood count, IL interleukin, Mo month, MSD mesoscale discovery, SEM standard error of the mean, SIV simian immunodeficiency virus.

Weight (kg) was measured in each monkey over the course of the study. We observed that Group 1 (untreated, maintained on ART) animals continued to lose weight during the study, whereas the EBT-001 treated groups kept on their trajectory of weight gain as is expected for their normal group (treated vs untreated, *P* = 0.029) with the highest dose animals having the largest weight gains (Fig. 5A). Among the cohort receiving the highest dose of EBT-001, neither animal experienced weight loss and both had increases in absolute lymphocyte count (Fig. 5B) without an increase in monocytes (Fig. 5C). No changes were seen in cholesterol, glucose, phosphorous, calcium, globin, and total protein in EBT-001 treated animals when comparing pre-infection, pre-EBT-001 to 3 months post-EBT-001 (Fig. 5D–I). However, the ART-only treated animal, IT6, had severe leukopenia (Fig. 5B), moderately severe hypophosphatemia (Fig. 5F), moderate hypocalcemia (Fig. 5G), severe anemia, and hypoproteinemia (Fig. 5H, I). In addition, untreated animal, 7S0, had marked hypoglycemia (Fig. 5E).

No notable changes in clinical chemistry or hematology parameters were observed in EBT-001 treated animals at pre-infection, pre-EBT-001, or 3 months post EBT-001 (Fig. 5J-L). However, the Day 6 post-EBT-001 dose laboratory evaluation of the 2 animals in Group 4 (highest dose) showed transient increases in serum concentrations of the liver enzymes, alkaline phosphatase (ALP), alanine transaminase (ALT) and aspartate aminotransferase (AST), and in total serum bilirubin (collectively referred to as liver function tests) (Fig. S7). Importantly, the elevated liver function tests returned to baseline range values in both NHP within 1 to 8 weeks (Table S10), and no evidence of liver necrosis or other signs of liver injury was observed by microscopic examination at the scheduled termination of the study at 6 months following dosing. Interferon (IFN)-gamma, interleukin (IL)-7, and IL-15 (Fig. 5M–O) cytokines were examined in plasma in animals after EBT-001 to check for adverse cytokine responses to AAV9 and immune responses. IFN-gamma was only elevated in one untreated animal and none of the EBT-001 treated group (Fig. 5M). IL-7, important in T cell development and HIV killing, was increased in one animal from Group 4 and IL-15, which activates natural killer (NK) cell-mediated viral responses, trended to increase in the treated animals (Fig. 5N–O).

### Off-target assessment using whole genome sequencing

The potential for unintended off-target editing in the EBT-001 treated NHPs was assayed in lymph nodes using whole genome sequencing analysis (WGS) to provide a means to identify possible unintended editing events across the genome. WGS was conducted on genomic DNA samples from lymph node biopsies collected from 2 NHPs, CK49 and CH97, before receiving EBT-001 and from the same animals after 3 months or 6 months of treatment with EBT-001, respectively. WGS was performed using 2 × 150 bp paired end sequencing and averaged ~30× coverage depth. To effectively align and process the sequencing reads, a pipeline was designed in the High-performance Integrated Virtual Environment (HIVE), a cloud-based environment optimized for storage and analysis of extra-large data, such as NGS (9; Supplementary text). When assessing for unintended off-target editing, we focused on the nominated sites with relatively higher homology to the guide sequences. The indel rates were calculated for genomic loci within 10 bp of hypothetical cut sites of each nominated locus. When we compared the treated with untreated samples from lymph node, the initial screen identified 5 sites to have significantly different numbers of SNPs (Table 2) using two-tailed chi-squared test with the Benjamini-Hochberg procedure to control false discovery rate (FDR). We observed that all these SNPs occur at a distant location from the hypothetical cut site (7–10 bases away) with SNPs showing in both treated and untreated samples. Further examination of these sites has shown a lack of evidence to attribute these changes to gene editing due to alignments being too noisy, having long stretches of Ts around the potential site that may result in ambiguous alignment, or coverage being low (Supplementary text). Additionally, it was noted that the relative change of the SNPs frequency was not consistent between the two select animals across all 5 sites. Hence, we conclude that there is lack of evidence for off-target editing activity. We also screened for structural variants among these nominated sites, we detected no read supporting partial alignments within 40 bp windows surrounding the nominated cut sites. Additionally, we looked at potential AAV integration events by searching for chimeric reads with partial alignment. Compared to samples before treatment, the analysis found only 2 potential reads in CH97 post-treatment and 1 read in CK49 post-treatment. The 2 reads from CH97 post treatment were identical to each other, possibly PCR duplicates, and showed partial alignment to Chr7:128862755–128862834 with partial alignment to Cas9 coding region (EBT001: 2636–2565). The 1 read in CK49 post treatment also showed partial alignment to Cas9 coding region (EBT001: 2944–3037), and partially aligned to Chr12:28738543–28738586 (Fig. S8). However, it was noted that among these three reads, none of their respective paired end read supported the partial alignment results. Taken together with the extremely little amount of supporting reads, we concluded that none of the 3 split-read jumps can be attributed as evidence for AAV integration in the lymph nodes after EBT-001 treatment.

**Table 2:** Sites identified with significantly different indels located within 10 bp of a nominated cut site in the WGS.

				Distance from cut site	Untreated		EBT-001		
Guide and nominated site ID	Monkey	Ref	SNP		Freq	Depth	Freq	Depth	Diff %
SIV_031_6MM_gag_03	CH97	G	+T	7	100%	19	95.8%	24	−4.2
SIV_031_6MM_gag_03	CK49	G	+T	7	68.0%	25	91.7%	24	23.7
SIV_052_4MM_ltr_13	CH97	T	+A	−10	35.9%	53	34.4%	96	−1.5
SIV_052_4MM_ltr_13	CK49	T	+A	−10	39.3%	56	43.5%	85	+4.2
SIV_093_5MM_ltr_44	CK49	A	+T	−10	65.6%	32	85.2%	27	19.6
SIV_093_5MM_ltr_44	CH97	A	+T	−10	95.2%	21	90.0%	10	−5.2
SIV_130_5MM_ltr_73	CK49	G	–	7	30.5%	584	26.4%	606	−4.1
SIV_130_5MM_ltr_73	CH97	G	–	7	24.7%	663	29.2%	634	+4.4
SIV_135_4MM_gag_03	CK49	A	+C	10	13.4%	739	11.5%	861	−1.9
SIV_135_4MM_gag_03	CH97	A	+C	10	12.3%	1023	13.0%	864	+0.7

Each location had a SNP rate compared to the reference genome in untreated and CRISPR-treated samples, which varied between NHP. The direction of change at each site alternates between the monkeys—a positive change is seen with one NHP, while a negative change is seen in the other—which is more consistent with sequencing noise, as seen in the alignments. Distance in nucleotides is given, as the SNPs are also not overlapping the nominated cut sites. Negative numbers are 5′ from the cut site.

*Diff* difference, *Freq*, frequency, *Ref* reference, *SNP* single nucleotide polymorphism.

## Discussion

Treatment of SIV-infected rhesus monkeys with AAV9 CRISPR-Cas9 and dual gRNAs targeting SIV LTR and Gag (EBT-001) exhibited broad tissue biodistribution and evidence of SIV proviral DNA editing in all major viral reservoirs. EBT-001 was well-tolerated at doses of 1.4 × 10^12^ GC/kg, 1.4 × 10^13^ GC/kg, and 1.4 × 10^14^ GC/kg. There were no clinical signs of toxicity and no abnormal gross pathology or organ histopathology attributed to EBT-001. One animal experienced an infusion related reaction thought to be due to anesthesia as the symptoms started after administration of anesthesia and before infusion of EBT-001, and another animal developed a rash at the infusion site. Transient increases in liver enzymes and total serum bilirubin were observed with the highest dose. Elevations in liver function tests following treatment with EBT-001 may represent an acute hepatic inflammation resulting from the treatment. Importantly, the elevated liver function tests returned to baseline range values in both NHP within 1–8 weeks. The transient nature of the elevations, combined with an absence of histopathological evidence of liver injury 6 months following treatment, indicate that the effects on liver function were reversible.

One interesting observation was the absence of weight loss among our SIV-infected cohort who received EBT-001, in contrast to the observation of weight loss seen in NHPs in the ART-only group. Interestingly, among our NHPs that were receiving the highest dose (1.4 × 10^14^ GC/kg) of EBT-001 and exhibiting no signs of weight loss, we also noticed improvements in their absolute lymphocyte counts within 6 months. Although we do not have data from untreated animals at 6 months, EBT-001 did not cause an elevation in IFN-gamma. Other cytokines, including IL-10, were examined but were either undetectable or no changes were seen. One animal in the highest dose group, BM79, had elevated IL-7 and IL-15 at 6 months, but without an SIV only control at this time point it is difficult to delineate if this is in response to AAV or SIV. In addition, the data suggests that EBT-001 may potentially benefit the immune system via lymphocyte recovery, T cell development, and NK cell maintenance.

One of the main concerns with the use of gene therapies in humans is their potential to induce immunological responses that may have adverse clinical effects [14–16]. These adverse effects may result from the presence of pre-existing antibodies to the AAV capsid, pre-existing antibodies to Cas9 [17], the formation of antibodies to the AAV capsid after vector treatment, the formation of antibodies to the transgene or its product, or from less specific immune responses [15]. The AAV antibody prevalence is similar in people with and without HIV and is similar to the prevalence in NHPs. AAV9 is one of the serotypes with the lowest frequency of pre-existing antibodies in the human population with ~33.5% of people with an AAV9 nAb titer [18, 19]. In our studies, increases in anti-AAV9 neutralizing antibodies were observed in the highest dosing groups (Group 3, 4) at 6 months following EBT-001 administration. We saw no evidence of ADCC-type attack on normal tissue in antibody-bearing animals and pathology was similar in all animals. SIV DNA excision was still evident, as observed in blood from individual animals at the earliest timepoint tested (2 weeks following EBT-001 administration) and up to 6 months following administration of the vector. In addition, neither monocytes nor IFN-gamma were increased in EBT-001-treated animals, which would have indicated an inflammatory response to EBT-001 or AAV9.

EBT-001 was well distributed to major organs and tissues that are identified as viral reservoirs of HIV, including the brain, an important reservoir for an HIV cure [20, 21]. Expression of EBT-001 DNA was detected in a dose-dependent and time-dependent manner. Overall, animals receiving the 10^12^ dose had lower log10 copies of EBT-001 DNA compared to the animals receiving the 10^13^ or the 10^14^ doses and had undetectable levels at the time of necropsy in some tissues, such as bone marrow compartment, brain, skeletal muscle, and testes. EBT-001 DNA levels in blood decreased over time as expected and EBT-001 DNA was only detected at the terminal bleed in animals treated with the 10^13^ dose and necropsied at 3 months post-infection. For HIV cure studies, sustained EBT-001 expression is not needed and the dose of AAV needed in the clinical setting is not yet known.

The pharmacologic gene editing activity of EBT-001 was demonstrated by the detection of SIV DNA excision, the removal of intervening DNA sequences between 5′LTR to Gag and/or Gag to 3′LTR, in organs and tissues tested at 3 and 6 months after vector administration. The 5G assay detects the amplicon bridging the excised region between 5′LTR and Gag; while the G3 assay detects the amplicon bridging the excised region between Gag and 3′LTR. Of note, there are 3 possible excision products that can result when using gRNAs targeting LTR and Gag. Due to the homology between the 5′ and 3′ LTRs, only 2 of the 3 excision are currently measurable. Thus, we are possibly underestimating the excision as we cannot detect evidence of the full 5′ to 3′LTR excision. However, the success of any one of the 3 possible excisions will render the viral unable to replicate.

With any gene editing platform, possible off-target effects are a concern that should be monitored. We mitigated this concern in this study using bioinformatics searches of the rhesus macaque genome that revealed no genomic sites output with 1, 2, or 3 mismatches to the guide and protospacer-adjacent motif (PAM). One site was identified for the LTR guide that had 2 mismatches plus a bulge. Few sites were output with 4 or 5 differences between the guides and the rhesus macaque genome (Table 1). Assays for possible off-target events for EBT-101 in HIV-1 infected humanized mice revealed no detectible activity after treatment with the guides targeting HIV [6]. Similar studies were conducted testing the SIV homolog EBT-001 in a small study of a single dose cohort of SIV-infected NHP [4]. To assay for possible unintended edits in this study, assays searched for indels and the possibility of larger changes, including insertions, deletions, inversions, translocations, or other chromosomal rearrangements. Past studies mapping the effects of CRISPR editing using a range of different gRNAs predominantly found single nucleotide deletions or insertions, a lower frequency of different small indels and a much lower frequencies of larger deletions and insertions [22, 23]. When larger deletions occurred, smaller indels were found at a higher frequency. Our analysis, therefore, included searching both for indels and possible larger changes. A marked advantage of the combination studies is the opportunity to use multiple pipelines simultaneously to assay for possible off-target editing and for the data from one pipeline to improve the accuracy of the data in other pipelines. For example, if structural changes or AAV insertion were detected at sites of off-target editing, these hypothetical events would be supported by evidence from indel analysis that were observed to occur at the same chromosomal sites with a higher number of sequence-confirmed indels. The simultaneous use of pipelines for indels, structural variation and AAV insertions can therefore help to assay for any possible, low-frequency editing sites. Here, WGS was performed without detecting unintended editing events and with no conclusive evidence of AAV or SaCas9 insertions, structural variations, or disruption of a gene, due to EBT-001 CRISPR editing. These NHP studies observed the intended SIV excision activity across multiple tissues studied with no detectible unintended editing.

This study was designed as a preclinical study for the safety and tolerability of EBT-001 in a large animal model of HIV. To most accurately model EBT-101, SIV guides were not optimized for cutting efficiency, but were instead selected to target the same LTR and Gag regions. Other than the guide sequences, EBT-001 and EBT-101 are comprised of an identical all-in-one delivery vector. The limitations of our study are the small animal numbers per group, the limited time on ART prior to EBT-001, and the lack of an analytical treatment interruption. The study was not designed to test the effect on the intact viral reservoir as EBT-001 was given when the viral reservoir was not stable and there was still active viral replication. Additionally, as animals were maintained on ART, we did not test the ability of EBT-001 to extend time to viral rebound or eliminate viral reactivation after an analytical treatment interruption. Together, the biodistribution and safety data support the continued development of AAV9 delivered CRISPR-Cas9 dual gRNA for HIV eradicative cure strategies.

### Supplementary information

Supplemental figures

## Data Availability

All data are available in the main text or the supplementary materials. Whole genome sequencing data is deposited to NCBI SRA under Accession PRJNA957550.
