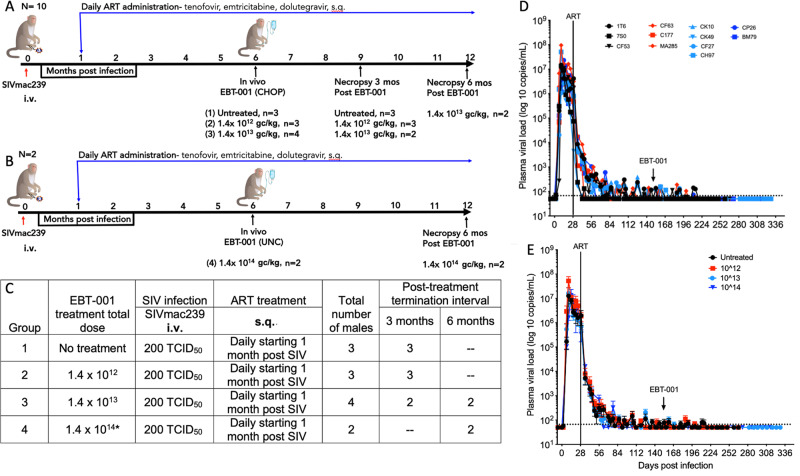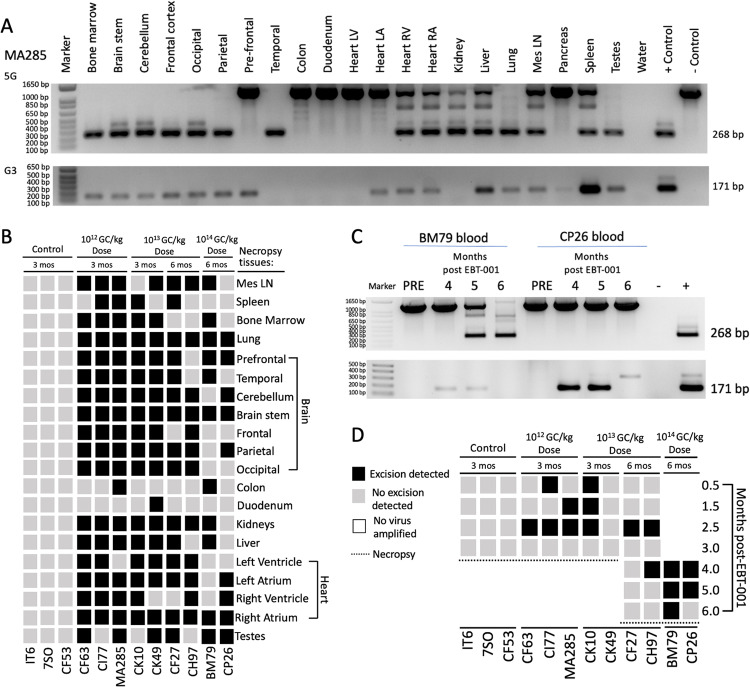# Correction: Preclinical safety and biodistribution of CRISPR targeting SIV in non-human primates

**DOI:** 10.1038/s41434-023-00438-6

**Published:** 2024-01-12

**Authors:** Tricia H. Burdo, Chen Chen, Rafal Kaminski, Ilker K. Sariyer, Pietro Mancuso, Martina Donadoni, Mandy D. Smith, Rahsan Sariyer, Maurizio Caocci, Shuren Liao, Hong Liu, Wenwen Huo, Huaqing Zhao, John Misamore, Mark G. Lewis, Vahan Simonyan, Elaine E. Thompson, Ethan Y. Xu, Thomas J. Cradick, Jennifer Gordon, Kamel Khalili

**Affiliations:** 1 Department of Microbiology, Immunology, and Inflammation, Center for NeuroVirology and Gene Editing, Lewis Katz School of Medicine, Temple University, Philadelphia, PA 19140 USA; 2 Excision BioTherapeutics, Inc., San Francisco, CA USA; 3 Center for Biostatistics and Epidemiology, Department of Biomedical Education and Data Science, Lewis Katz School of Medicine, Temple University, Philadelphia, PA 19140 USA; 4 BioQual, Inc., Rockville, MD USA; 5 Embleema, Metuchen, NJ USA

**Keywords:** Virology, Clinical genetics

Correction to: *Gene Therapy* 10.1038/s41434-023-00410-4, published online 17 August 2023

In this article, the 17th author’s name, Elaine E. Thompson was missing from the author list and the author contribution texts. The author contributions should have read as follows.

Revised Fig. 2

**AUTHOR CONTRIBUTIONS**

Conceptualization: THB, JG, KK. Methodology: CC, RK, PM, MD, RS, MDS, MC, SL, HL, WH, VS, EET, EYX, TJC, HZ, IKS, JM. Writing—original draft: THB, KK. Writing—review & editing: KK, IS, JG, TJC, WH, MGL. Interpretation: THB, KK, JG, TJC, HZ, RK, IKS.

Revised Fig. 4

Figure 2 and Figure 4 had unnecessary red lines and Figure 4 images were partly cut off. The errors resulted from an error during manuscript submission. Please note that they do not affect the data presented in the figures and do not impact the results or conclusions of the paper; the figures should have appeared as shown above.

The original article has been corrected.