# Supplementary material for: Preclinical safety and biodistribution of CRISPR targeting SIV in non-human primates
Source: Gene Ther. 2023 Aug 17;31(5-6):224–33. doi: 10.1038/s41434-023-00410-4 (PMC11090835; doi:10.1038/s41434-023-00410-4)
Supplement: Supplementary file 1 — Supplemental figures [file 41434_2023_410_MOESM1_ESM.pdf]

May 1, 2023

SUPPLEMENTAL MATERIAL

Materials and Methods:

Computational nomination of off-target editing sites in the rhesus macaque genome:

COTANA (CRISPR Off-TArget Nomination & Annotation) was used to nominate and direct the assays for possible unintended editing at the nominated chromosomal locations. COTANA is based on the search algorithm of Cas-OFFinder (1) and uses customized post processing to correct, score and rank the nominated sites. The binaries of Cas-OFFinder 2.4.1 were downloaded from Github (https://github.com/snugel/cas-offinder/releases, on October 28, 2021) and deployed in Excision BioTherapeutics' internal Amazon Web Services (AWS) cloud computing environment. The rhesus macaque reference genome searches used the latest NCBI genome assembly Mmul_10 (https://www.ncbi.nlm.nih.gov/assembly/GCF_003339765.1/) with SaCas9-specific PAM sequence: 5′-NNGRRN-3′, allowing up to 5 mismatches and 1 bulge. COTANA further postprocessed and summarized the results. First, the loci from the alternative chromosomes were removed. The PAM location for each nominated locus was computed. The results were de-duplicated. Next, local re-alignment was conducted for each predicted locus against its guide sequence, and all possible alignments were generated. Each possible alignment was scored using our customized SaCas9 position weight matrix (**Table S1**). The alignment with the lowest score was processed as the most probable one, from which the number of mismatches and bulges was computed and reassigned to the locus. Finally, genomic loci with the total number of differences of 5 (up to 4 mismatches with 1 bulge or 5 mismatches with 0 bulge) were categorized in **Table 1**, and the alignments provided (Supplemental Datasets 1 and 2). The alignments from selected genomic loci were shown in **Figure 1**. RNA Bulge: bulge introduced in the guide sequence during alignment. DNA Bulge: bulge introduced in the target DNA sequence during alignment.

EBT-001 dose and formulation: EBT-001 (AAV9-SaCas9.U6sgLTR-1.U6sgGagD) was manufactured by recombinant technology using the mammalian host cell line, HEK293, which is transfected with 3 plasmids: AAV9 packaging plasmid, adenovirus helper plasmid, and plasmid containing 2 gRNAs targeting the SIV long terminal repeat (LTR) and the group specific antigens (Gag) gene (2). This is the same all-in-one construct that we have used in our prior studies, which encodes both gRNAs and SaCas9 (2). EBT-101 was formulated as a sterile drug product in compendial excipients by Clinical Vector Core, Center for Cellular and Molecular Therapeutics, Children's Hospital of Philadelphia (CHOP). For the n = 10 study (lot A9FP1-C1806D concentration of 1.14 x 10^{13} vg/mL), EBT-001 was diluted in 100 mLs of 1X phosphate buffered saline (PBS). For the 1.4 x 10^{12} dose, 0.5 – 0.6 mL of EBT-001 was added to 100 mL of PBS prior to IV infusion. For the 1.4 x 10^{13} dose, 5.3-6.4 mL of EBT-001 was added to 100 mL of PBS prior to IV infusion. For the n = 2 study, the plasmid was packaged into AAV-9 at University of North Carolina Vector Core (lot LAV102ABCD concentration of 1x10^{13} vg/mL), The amount of vector to be dosed was added to total of 100 mL of 1X PBS for each animal based on current body weight. For group 4, 54.6 or 53.2 mL of EBT-001 was added to 45.4 or 46.8 mL of PBS prior to IV infusion (**Table S2**). A single infusion was administered at an infusion rate of 1 mL/min with Benadryl IM 2 mg/kg administered 30 minutes prior to the procedure and then another dose IV immediately prior to the EBT-001 infusion.

qPCR for plasma viral load: The assay utilized primers and a probe specifically designed to amplify and bind to a conserved region of the Gag gene of SIV. The signal was compared to a known standard curve and calculated to give copies per mL or per 10^6 cells. A volume of 0.2 mL of the ethylenediaminetetraacetic acid (EDTA) plasma was used and added to 0.2 mL of buffer with carrier RNA. The sequences for the SIV primer/probe sets are as follows: SIV-Forward primer: GTCTGCGTCATCTGGTGCATTC, SIV-Reverse primer: CACTAGGTGTCTCTGCACTATCTGTTTTG, Probe: SGAG23: 6FAM-CTTCCTCAGTGTGTTTCACTTTCTCTTCTGCG–TAMRA. To make the RNA control, stock SIV

was processed following the same procedure. RNA controls: To make the RNA control, stock SIV was processed following the same procedure described above. To determine the amount of RNA, an optical density (OD) reading at 260 was taken: 1.0 OD at A260 = 40 µg/mL of RNA. With the number of bases known and the average base of RNA weighing 340.5 g/mole, the number of copies was then known, and the control was diluted accordingly. The final dilution of 10^7 copies per 3 µL was divided into single use aliquots of 10 µL. These were stored at –80°C until needed. Several aliquots were chosen at random and compared to previous controls to verify consistency. 2.5 mL of 2X buffer containing Taq-polymerase obtained from the TAQMAN RT-PCR kit (Bioline USA Inc., Boston, MA) was added to a 15 mL tube. Then, 50 µL of the RT and 100 µL RNAse inhibitor from the kit was added. The primer pair at 2 µM concentration was also added in a volume of 1.5 mL. Lastly, 0.5 mL of water and 350 µL of the probe at a concentration of 2 µM were added, and the tube was vortexed. Reactions: 47 µL of the master mix and 5 µL of the sample RNA were added to each well in a 96 well plate. All samples were tested in triplicate and the plates were sealed with a plastic sheet. Samples of the control RNA were obtained from the –80°C freezer. The control RNA has been prepared to contain 107 copies per 3 µL. Eight 10-fold serial dilutions of control RNA were prepared using RNAse-free water by adding 5 µL of the control to 45 µL of water and repeating this for 6 dilutions. This resulted in a standard curve with a range of 1 to 10^6 copies/reaction. Triplicate samples of each dilution were prepared as described in the beginning. The sample was diluted as needed if the copy number exceeded the upper limit of detection. The plate was placed in an Applied Biosystems 7500 Sequence detector and analyzed using the following program: 48°C for 30 minutes, 95°C for 10 minutes followed by 40 cycles of 95°C for 15 seconds and 1 minute at 60°C. A printout of the results was maintained in the laboratory notebook. The number of copies of RNA per mL was calculated by extrapolation from the standard curve and multiplied by the reciprocal of 0.5 mL extraction volume, i.e. 20. This gave a practical range of 50 to 5 x 10^7 RNA copies per mL. The 7500 Sequence detector is calibrated at least annually by Applied Biosystems. The intra assay variation of this test was around 0.3 Log.

Necropsy: After terminal blood and sample withdrawal under ketamine/acepromazine anesthesia, all main study animals euthanized (Beuthanasia 0.1 ml/kg IV until cessation of respiration but not heartbeat; thoracotomy with insertion of 16-gauge needle into left ventricle and begin flow of heparinized NaCl; open right atrium and remove expelled blood/NaCl mixture under suction; continue perfusion until aspirated fluid is clear [generally 1 to 1.5 liters]) and subjected to a complete necropsy examination. All tissues for biodistribution were snap frozen and stored at -80 until shipment to Charles River Laboratories. The tissue samples collected at necropsy and designated for biodistribution analysis were transferred to the Cellular and Molecular Biology lab at Charles River and analyzed by qPCR. Tissues for paraffin blocks were placed in 4% paraformaldehyde for 24 hours then transferred to alcohols and then sent to American Histopathology for embedding. The tissue samples collected at necropsy and designated for histopathology, were stored in 10% NBF and transferred to the Charles River Pathology Associates International, Frederick, MD and analyzed for histopathological changes. Samples collected for qPCR analysis were collected using strict aseptic techniques. Care was given to ensure cross contamination between tissues did not occur. Gloves were changed between collection and dissection of each tissue. In addition, non-disposable instruments were wiped down with a 10% bleach solution, rinsed with water, and followed by a wipe down of 100% ethanol between each group. Tissues were collected from control animals prior to collection of tissues from the EBT-001-treated groups. Experimental Pathology Laboratories, Inc. was responsible for good laboratory practice neuropathology assessment (study number C63-002).

Vector expression (DNA) in serial blood samples: DNA from NHP blood (200uL) was isolated using a NucleoSpin Tissue kit (Macherey-Nagel). Digital droplet PCR (ddPCR) was performed based on the water-oil emulsion droplet technology, using the ddPCR™ Supermix for Probes reagents in the QX200™ Droplet Digital™ PCR system (Bio-Rad Laboratories, Hercules, CA, USA). For quantification of AAV vector, genomic DNA was PCR amplified using TaqMan set targeting the SaCas9 AAV transgene and rhesus macaque TERT gene as a reference. A total of

50-100ng DNA from blood was used as template for ddPCR amplifications under the following conditions: 95℃, 10 minutes; 44 cycles: 95℃ 30s, 57℃ 60s, 72℃ 60s; 98℃ 10 minutes; 12℃. Data were analyzed using QuantaSoft (Bio-Rad). For the AAV vector saCas9 transgene, the sequence of the forward primer (saCas9 F 1799-1818) is 5′-CGACATCAAGGACATTACCG-3′, the reverse primer (saCas9 R 1873-1882) is 5′-GCTGCTCTGGTAGATGGTCA-3′, and the probe (saCas9 probe FAM 1839-1858) is 5′-AACGCCGAGCTGCTGGATCA-3′. For the rhesus macaque DNA reference gene TERT, the forward primer (RmTERT F Ch6 1158407-1158427) 5′-GAGCTGAGATTGTGCCCTTG-3′, the reverse primer (RmTERT R Ch6 1158579-1158598) is 5′-CCATTTGCTGTCCTCTGCTC-3′, and the HEX probe (RmTERT probe HEX Ch6 1158531-1158552) 5′-CCAGCACAGATCCTGGTCCCGT-3′.

saCas9 DNA expression: A TaqMan-based qPCR assay was validated for absolute quantitation of EBT-101 vectors in dosed NHP (Charles River). The assay's amplicon is specific to EBT-101 vector DNA in the background of rhesus monkey gDNA. Total DNA was extracted from rhesus macaque monkey tissue and blood using QIAsymphony® DSP DNA mini kit. QIAsymphony. qPCRs were performed in 96-well plates. Ever run had a standard curve, 2 sets of quality control (QC) samples, in addition to the study samples. The standard curve was run in 3 replicates for the standard points at 10^7, 10^5, 10^4, 10^3, 10^2, 50, 25, and 0 copies per reaction. The 0 copy standards, containing only matrix, were included to confirm reagent purity. Duplicate QC samples were prepared at 10^6 (QC-H), 10^4 (QC-M), and 10^3 (QC-L) copies/reaction in a background of 1,000 ng monkey matrix DNA per reaction. Tissue DNA samples were tested at 1,000 ng per reaction, and blood was tested at 100 ng per reaction. All sample reactions were run in triplicate, and the third reaction was spiked with 200 copies of px601 DNA to evaluate potential qPCR inhibition. The forward primer used is 5'-CGACATCAAGGACATTACCG-3', and the reverse primer used 5'-GCTGCTCTGGTAGATGGTCA-3'. The probe was 5'-AACGCCGAG/ZEN/CTGCTGGATCA-3'. The lower limit of quantitation of the target DNA was 25

copies of px601 DNA or dsEBT-001 vector gDNA per reaction. The lower level of detection (LOD) was 10 copies of px601 DNA or ds EBT-001 vector gDNA per reaction.

EXA assays: Extensive blood and tissue collection was performed for assessment of excision of SIV DNA at study termination. Blood and tissues were processed, DNA extracted, and PCR analysis run for determination of excision products. Genomic DNA were extracted from tissues using DNA extraction kit (Macherey-Nagel, Cat No./ID: 740952.250) according to the manufacturer's instructions for tissue extractions. All excision assays were performed with Failsafe Enzyme Mix and Buffer D (Epicentre #FS99100). The assays provided qualitative assessment of the 2 excision amplicons/products extracted from the internal organ and tissues samples to the validated LOD of one copy per 250ng genomic DNA samples. The 5G excision assay consisted of three-step sequential double-nested PCR reactions for 5′-LTR to Gag, refer to the gRNAs and excision assay primers used for 5G EXA (**Table S3**). The excision assay for G3 EXA consisted of three-step sequential double-nested PCR reactions for Gag to 3′LTR regions, refer to the gRNAs and excision assay primers used for G3 EXA (**Table S3**). The unexcised viral amplicon in G3 EXA was too large to be amplified using this extension time. Therefore, there were no negative control for unexcised viral DNA used for G3 EXA assays. The bioanalytical validation results demonstrated the assay precision with a limit of detection of one (1) copy per reaction.

5′LTR-gag excision efficiency calculations: Jpeg agarose gel images were converted to grayscale and analyzed using the ImageJ gel analysis tool (3). The profile plots were generated for each line of the agarose gels, and areas of the peaks corresponding to the top (unexcised), and bottom (excised, CRISPR-cleaved/end-joined) bands were measured and then converted in Microsoft Excel into percent excision using the formula: [bottom (excised) band peak area/top (unexcised) + bottom (excised) bands peak areas)] × 100%. Bands that were higher or lower than the expected full-length size were sequenced, and these unspecific bands were not taken into consideration in the calculations.

Whole genome sequencing via the high-performance integrated virtual environment (HIVE) pipeline: Cells were harvested, and the whole genome was sequenced at an average depth of around 30x. The cloud-based HIVE pipeline was designed for the storage and analysis of extra-large datasets. The HIVE-hexagon sequence aligner was chosen due to its ability to generate accurate alignments even for regions that are highly divergent from reference genome (4). Data loading was initiated for all paired-end reads, followed by parsing, format validation, indexing, and storage of metadata in the HIVE Honeycomb database. Next, a variety of QC and screening computations were performed in the QC pipeline. For these NHP studies, whole genome sequencing (WGS) reads were aligned to the rhesus macaque reference genome Mmul_10 and to dummy chromosomes corresponding to the AAV and to the SIV reference genome (SIVmac239). Searching for either AAV insertion or structural changes requires detecting sequencing reads that map to more than one location in the reference genome or additional sequences and weighing the evidence for each partial alignment. Any reads aligning to multiple regions (split reads) were selected from the pools, and their paired ends analyzed using DI Profiler. To detect split reads between AAV and the genome, the reads that aligned to the AAV sequence were collected and aligned simultaneously to the genome and AAV sequence with the same parameters as before. The resulting alignments were evaluated for split reads with HIVE-DI-profiler, a tool that can map reads to more than one reference genome. The process was simultaneously done searching for SIV split reads and reads mapping to more than one genomic location. DI Profiler was run requiring only a minimum depth of 1 read to capture all split reads d in the output. This process was parametrized to detect partial "sequence jumps" of at least 30 nucleotides on each reference genome. Data loading of all dual index demultiplexed FASTQ files was initiated for all paired-end reads, followed by parsing, format validation, indexing, and storage of metadata in the HIVE Honeycomb database. After data loading, a sequence QC pipeline that performs a variety of different QC and screening computations was automatically run (**Figure S1**).

Pipeline for the analysis of possible off-target Single Nucleotide Variation (SNV) and insertions or deletions (indels): HIVE-hexagon DNA sequence alignments were generated for each sequencing run. It was parameterized to detect both small and big insertion/deletion (indel) gaps of up to 28 nucleotides (twice the alignment seed size). The HIVE-heptagon variant caller performs SNV indel analysis by piling up the mapped sequences for each reference position and calling the frequencies for each type of event observed (5). All events were filtered using the knowledge of sequencing and index hopping noise, as well as alignment noise, due to homologies in repetitive genomic regions, coverage inhomogeneity, and reported polymorphisms. Careful examination of all events that have been observed in both treated and untreated samples have been attributed to one of the sources of noises described above. Other events that pass the abovementioned noise filters have been examined additionally for the correct variation of directionality, where edited samples are expected to have higher frequency of editing-related events compared to unedited samples. The leftover, correctly directional, differential events have then been analyzed for statistical significance between untreated control samples and test samples using Benjamini–Hochberg procedure to control the False Discovery Rate (FDR) using modified Bonferroni correction for multiple hypothesis testing (6). Editing effects are evaluated at the putative sites using a combination of knowledge-based filtration and statistical methods (**Figure S2**).

Pipeline for the analysis of possible structural changes: WGS short reads were aligned to the rhesus macaque reference genome Mmul_10 and to constructs corresponding to the AAV and to the SIV reference genomes (SIVmac239). All reads or read-pairs that were aligned along the entire read length to multiple reference locations have been force-mapped to a position with the highest alignment score. In contrast, the reads that have been aligned partially with different, non-overlapping parts of single reads are mapped either to different reference locations or chimerically, where a forward and reverse paired-end reads have been aligned to different chromosomal locations, which are processed separately (**Figure S3**). These reads are then

examined for potential structural alterations using DI Profiler (7). Partial and chimeric reads are called “split reads,” and non-overlapping read alignments to multiple locations are referred to as “sequence jumps.” Sequence jumps that involve AAV and host genome were assessed as potential AAV integration sites; jumps that involve different locations of SIV have been assessed as evidence of on-target double excision candidates: mappings that involve different host chromosome regions have been examined on a subject of potential off-target structural alterations. DI Profiler has been parametrized to require only a minimum depth of 1 read with at least 30 nucleotide mapping on each side to capture and process all confidently aligned sequence jumps. As for SNV and indel detection, all sequence jump events have been filtered against the noise and thresholds have been set to cut off anything below the demultiplexing errors and to filter ambiguous alignments into repetitive regions. A validation step has been performed to ensure and consider only events where edited samples are expected to have higher frequency of editing-related events compared to unedited samples. As a last stage, all differential events have been analyzed for statistical significance between untreated control samples and test samples using Benjamini–Hochberg procedure to control the FDR using modified Bonferroni correction for multiple hypothesis testing (6).

Pipeline for the analysis of possible AAV insertion: An analysis pipeline to detect possible AAV insertions and map insertion sites was developed that searches for split reads containing regions aligning to EBT-001 or SIV (mac239). Reads with partial alignments to EBT-001 or SIV are selected and aligned to the whole rhesus macaque reference genome alongside EBT-001 and SIV to determine if the reads are spanning a putative insertion site, either into the genome or in treated animals into the SIV sequences (**Figure S4**). To account for similarities between the rhesus macaque and human genomes, analysis for partial alignments in these regions is necessary. However, these alignments can easily be identified by their occurrence in both treated and untreated samples.

Analysis of lymph nodes from NHPs pre- and post-treatment with EBT-001: WGS was conducted on genomic DNA samples harvested from lymph node tissues of SIV-infected NHPs receiving daily antiretroviral therapy administration before and after treatment with EBT-001. Lymph node biopsies were collected from two NHPs, CK49 and CH97, before receiving EBT-001. Lymph nodes were then collected from the same animals after treatment with EBT-001 at the time of euthanasia. NHPs CK49 and CH97 each received a single intravenous injection of 1.4×10^{13} vg/kg and were euthanized at 3 months (CK49) and 6 months (CH97) after EBT-001 treatment. Both animals showed evidence of excision of SIV proviral DNA in most tissues tested at necropsy and were further studied for evidence of possible unintended edits. Genomic DNA was prepared in parallel from lymph nodes collected from NHPs CK49 and CH97 before and after EBT-001 treatment for a total of 4 samples. WGS was performed at 30x depth comparing the matched treated and untreated samples to minimize the difference between individual monkeys. The sequences were assayed for possible indels within 10 bp of the nominated cut site at each locus. Significance was assessed using a two-tailed chi-squared test on the number of reads, using the untreated values as expected and treated values as observed. P values were corrected for a FDR of $P<.05$ using the method of Benjamini and Hochberg. The 2 treated samples were compared to the WGS from the 2 untreated lymph node tissue samples using HIVE pipelines. A file of 206 reference sequences 1001 bp (~ 500 bases of flanks around cut site) in length were constructed from the Mmul_10 reference genome to use in analysis. Sequences were aligned to the nominated sites with HIVE-hexagon requiring a minimum of 75 aligned bp, with 5% mismatch to allow for alignments through potential indels.

Supplemental data:

AAV9 neutralizing antibodies: Neutralizing antibodies to AAV9 in HEK293 cells were measured in EDTA plasma prior to the study start (pre-dose) and at necropsy at the University of Pennsylvania, Gene Therapy Program, Immunology core using their standard operating

procedure. The results are summarized in **Table S4 and S5**. Three to 6 months after treatment, EBT-001 was associated with increases in AAV9 neutralizing antibodies relative to pre-dose values. The pre-dose titers (serum reciprocal dilution values) were all low (<5 to 80), except CF63 which had a pre-dose titer of 320. The values at necropsy ranged from 80-5120 in EBT-001 treated animals and were highest in the monkeys sacrificed at 6 months post-EBT-001. Of note, CF63's titer did not change after EBT-001 treatment.

Biodistribution of EBT-101 in the blood: Analysis by ddPCR of Cas9 transgene DNA levels in genomic DNA extracted from NHP blood at week 2, 5, 6, 9, 11, and 14 post-EBT-001 (**Figure S5)**. The mean (and standard error) Log10 copies of EBT-001 DNA per μg of monkey genomic DNA was graphed for animals in Group 1 (untreated), Group 2 (red, 10^{12}), and Group 3 (light blue, 10^{13}, 3 and 6 months). All samples were run in duplicate. Blood from Untreated group 1 were all below the LOD of the assay.

5G Excision efficiency in EBT-001-treated rhesus macaques: Since the 5G EXA amplifies both the full-length virus and the amplicon of the excision product, we can qualitatively calculate a percent efficiency of the 5G excision (**Figure S6**, **Tables S8, and S9, Supplemental material and methods**). Of note, this is only one of 3 possible excision products that can occur through EBT-001 cutting so not a reflection of the total efficiency. As seen in **Tables S6 and S7** and as shown in **Figure 4**, excision may have been detected in the G3 EXA but not in the 5G EXA and vice versa, but either way inactivates the virus.

Liver function tests: No notable changes in clinical chemistry or hematology parameters were observed except for transient increases in serum concentrations of the liver enzymes, alkaline phosphatase (ALP), alanine transaminase (ALT) and aspartate aminotransferase (AST), and in total serum bilirubin (collectively referred to as liver function tests) at the first post-dose laboratory evaluation at Day 6 (**Figure S7**). In the animal (CP26) with the higher ALT and AST elevations, a transient loss of appetite was observed for 2 days post-dose but returned to normal the following day. No other clinical signs were observed in these 2 NHP. The elevated liver function tests returned to baseline range values in both NHP within 2 to 9 weeks (13-62 days) post-dose or 1 to 8 weeks following observed peak values on Day 6 (**Table S10**). At the scheduled termination of the study 6 months following dosing, no evidence of liver necrosis or other signs of liver injury were observed in either animal by microscopic examination of tissues. Due to the transient nature of the liver enzyme changes, and lack of microscopic correlates indicative of liver injury at necropsy, these findings are not considered adverse.

AAV insertion analysis of EBT-001-treated rhesus macaques: To address the possibility of AAV9 integration, sequence reads were monitored for vector insertions by utilizing pipelines to identify sequence reads with homology to the AAV vectors and pipelines to search for evidence of genomic integration. This work was coordinated with our assays for possible off-target editing, as published works link AAV integration to genomic sites with identified nuclease cleavage activity. Exhaustive bioinformatic searches using the SIV guides did not identify sites with high probability match scores in the rhesus macaque genomes (Table 1). In fact, only 1 chromosomal site was found with 3 or less differences with either guide (Figure 1). The likelihood of viral integration greatly decreases without detectible genomic cleavage events. Conversely, increasing off-target genomic breaks leads to increased AAV insertion. As DNA breaks happen in cells with and without treatment, AAV integration, as well as transfected oligonucleotide integration, can be seen without nuclease-induced DNA cleavage (8), WGS was used to survey for changes genome-

wide and to search for possible AAV integration using a pipeline assaying for split reads linking viral and genomic sequences.

The analysis found only 2 potential reads in CH97 post-treatment and 1 read in CK49 post-treatment. The 2 reads from CH97 post treatment were identical to each other, possibly PCR duplicates, and showed partial alignment to Chr7:128862755-128862834 with partial alignment to Cas9 coding region (EBT001: 2636-2565). The 1 read in CK49 post treatment also showed partial alignment to Cas9 coding region (EBT001: 2944-3037), and partially aligned to Chr12:28738543-28738586. The split reads in CK97 are likely due to a coincidental homology to a small portion of Cas9 in the genome. The other 2 sites were disqualified due to lack of reliable unique alignment because of the presence of long stretches of polyA/polyTs that may result in potential partial alignments in too many places in the genome of CK49, and none had good quality. Additionally, each detected sequence jump had marginally low coverage and were irreproducible across different animal samples, CK97 and CK49. Therefore, the conclusion was made that none of the 4 split read jumps can be attributed as evidence for AAV integration.

Assaying for possible off-target editing in EBT-101-treated rhesus macaques: Indel rates were compared between treated and untreated genomic sequences within 10 bp of the cut site at each locus. Significance was assessed using a two-tailed chi-squared test on the number of reads, using the untreated values as expected and treated values as observed. P values were corrected for a FDR of $P < .05$ using the method of Benjamini and Hochberg. A total of 17 sites in total that passed filters were tested for significance of their indel rate. The noise in the system was demonstrated by 12 of the sites having more miscalls in the untreated. The other 5 sites had greater sequencing variant reads in the treated cells, though manual examination showed a lack of evidence to attribute these changes to gene editing, due to alignments that were too noisy, despite passing threshold. There were long stretches of poly Ts around the potential site that may result in ambiguous alignment: putative "editing" changes were not at the cut site or coverage was

marginally low. Additionally, it was noted that none of the sites were consistent among different test/control pairs and were only observed sporadically for a single experiment with no reproducibility across different samples.

Structural analysis of EBT-001–treated rhesus macaques: WGS reads from both treated and untreated lymph node samples were aligned to the EBT-001 sequence, the SIV reference genome (SIVmac239), and to the rhesus macaque genome (Mmul_10) to search for split reads aligning to more than one location. Split reads were processed in the pipeline for discovery of off-target SNVs, indels, structural alterations, and AAV integrations and compared to reference constructs made of windows around the 206 nominated rhesus macaque genomic locations using the algorithm parameterized to detect partial alignments of up to 40 bases long with up to 5% mismatches. The resulting alignments were passed to the HIVE DI-profiler to detect split reads with partial alignment. No partial alignments were detected anywhere in the region of the nominated cut site ±20 base pairs. The few potential partial alignment split reads that were at a significant distance from the cut site are a likely result of homology between different nominated regions. In summary, there was no evidence in this experiment for structural variations between any of the nominated sites in these EBT-001 treated rhesus macaques.

Supplemental references

1. Bae S, Park J, Kim JS. Cas-OFFinder: a fast and versatile algorithm that searches for potential off-target sites of Cas9 RNA-guided endonucleases. *Bioinformatics.* 2014;30(10):1473-1475.
2. Mancuso P, et al. CRISPR based editing of SIV proviral DNA in ART treated non-human primates. *Nat Commun*. 2020;11(1):6065.
3. Schneider CA, Rasband WS, Eliceiri KW. NIH Image to ImageJ: 25 years of image analysis. *Nat Methods*. 2012;9(7):671-675.
4. Simonyan V, et al. High-performance integrated virtual environment (HIVE): a robust infrastructure for next-generation sequence data analysis. Database (Oxford) 2016;2016.
5. Simonyan V, Chumakov K, Donaldson E, et al. HIVE-heptagon: A sensible variant-calling algorithm with post-alignment quality controls. *Genomics.* 2017;109(3-4):131-140.
6. Feser WJ, et al. Calculating average power for the Benjamini-Hochberg procedure. *J Stat Theory Appl*. 2009;8(3):325-352.
7. Bosma TJ, et al. Identification and quantification of defective virus genomes in high throughput sequencing data using DVG-profiler, a novel post-sequence alignment processing algorithm. *PLoS One*. 2019;14(5):e0216944.
8. Tsai SQ, et al. GUIDE-seq enables genome-wide profiling of off-target cleavage by CRISPR-Cas nucleases. *Nat Biotechnol.* 2015;33(2):187-97.

TABLES:

Table S1. SaCas9-specific position weight matrix for the scoring of local re-alignments during the postprocessing of off-target sites nominated by COTANA to provide the Penalty Match Score. The Penalty Match Score for a perfect match is zero and increases with mismatches and bulges between the chromosomal DNA and the gudie RNA or PAM preference. The score added increases, as shown in the Table, moving closer to the PAM. The Penalty Match Score increases for bulges by adding 0.51 for RNA bulges and 0.70 for DNA bulges in addition to the position-specific value from the table. There is an additional score added (0,5) for adjacent mismatches or bulges.

Guide sequence	Position	Score for mismatch or bulge
Protospacer	-21	0.1
	-20	0.1
	-19	0.12
	-18	0.13
	-17	0.15
	-16	0.17
	-15	0.19
	-14	0.21
	-13	0.23
	-12	0.27
	-11	0.35
	-10	0.5
	-9	0.7
	-8	0.8
	-7	1.1
	-6	1.3

	-5	1.9
	-4	2.3
	-3	3
	-2	4
	-1	5
	0	6
N	1	0
N	2	0
G	3	40
R	4	20
R	5	20
T	6	2

Table S2. Animal date of birth, total viral genomes given and volume of EBT-001 stock.

***** CK49 received ~1/2 of EBT-001 split across 2 days, 4 days apart. CHOP, Children's Hospital of Philadelphia, N/A, not applicable; UNC, University of North Carolina; vg, viral genomes.

	**Animal number**	**Date of birth**	**Total vg given**	**Volume of EBT-001 stock, Lab**
Group 1 untreated 3 months	1T6	8/17/2014	N/A	N/A
	7S0	10/25/2014	N/A	N/A
	CF53	7/28/2015	N/A	N/A
Group 2 1.4 x 10^{12} GC/kg, 3 months	CF63	6/6/2015	6.02 x 10^{12}	0.5 mL, CHOP
	CI77	6/29/2016	6.02 x 10^{12}	0.5 mL, CHOP
	MA285	4/23/2015	6.58 x 10^{12}	0.6 mL, CHOP
Group 3 1.4 x 10^{13} GC/kg, 3 months	CK10	7/6/2015	6.16 x 10^{13}	5.4 mL, CHOP
	CK49	7/29/2015	7.14 x 10^{13}	6.3 mL, CHOP
Group 3 1.4 x 10^{13} GC/kg, 6 months	CF27	6/24/2015	7.28 x 10^{13}	6.4 mL, CHOP
	CH97	6/3/2015	6.02 x 10^{13}	*5.3 mL, CHOP
Group 4 1.4 x 10^{14} GC/kg, 6 months	BM79	2/13/2017	5.46 x 10^{14}	54.6 mL, UNC
	CP26	5/11/2017	5.32 x 10^{14}	53.2 mL, UNC

Table S3. 5G and G3 EXA primer sequences used in double-nested PCRs reactions and location of the gRNAs and primers.

PCR, polymerase chain reaction.

Assay	Primer name	Sequence
5G EXA	SIVmac239/ltr/-439/F1	GGCAGGATTACACCTCAGGA
	SIVmac239/gag/+117/R1	TCTGCAGCCTCCTCGTTTAT
	SIVmac239/nestedLTR/F2/3	AGACATTTGGCTGGCTATGG
	SIVmac239/nestedgag/R2	GGGTGCAACCTTCTGACAGT
	SIVmac239/nestedgag/R3	GTTTCTGTTGTTCCTGTTTCCACCACT
G3 EXA	SIVmac239/pcr/+595/F1	CTACGACCCAACGGAAAGAA
	SIVmac239/+9516/R1	ATTTTCCTGCTTCGGTTTCC
	SIVmac239/nested/+731/F2/3	CATTAGTGCCAACAGGCTCA
	SIVmac239/nested/+9734/R2	CACCCAGGCTCTACCTGCTA
	SIVmac239/nested/R3	AGCGAGTTTCCTTCTTGTCAGCCA

SIV 5'-LTR1-GagD Excision

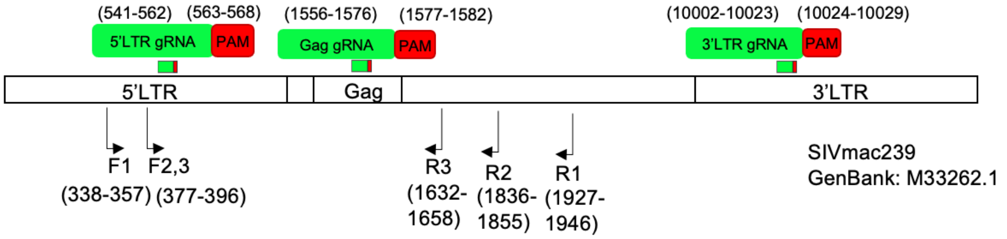

SIV GagD-3'LTR1 Excision

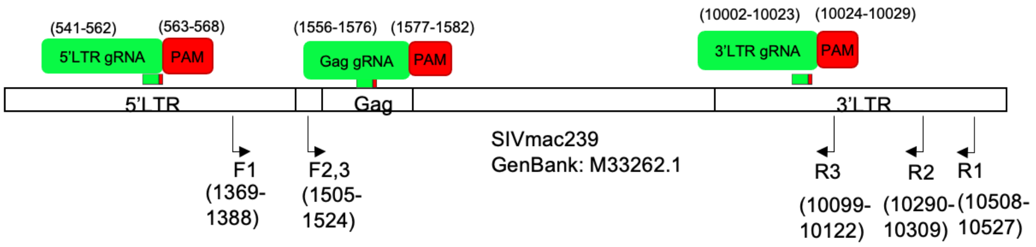

Table S4. AAV9 Neutralizing antibody titers

Values are the serum reciprocal dilution at which RLUs was reduced 50% compared to virus control wells (no test sample). Limit of detection 1/5 dilution.
AAV9, adeno-associated virus serotype 9; GC/kg, genome copies per kilogram; RLU, relative luminescence units.

Dose group	Animal number	Sampling timepoint	
		Pre-dose	Necropsy (months)
Group 1- control	IT6	80	640 (3 months)
	7S0	80	5 (3 months)
	CF53	<5	<5 (3 months)
Group 2- (1.4e12 GC/kg)	CF63	320	320 (3 months)
	CI77	10	80 (3 months)
	MA285	40	640 (3 months)
Group 3- (1.4e13 GC/kg)	CF27	10	2560 (6 months)
	CH97	10	2560 (6 months)
	CK10	5	320 (3 months)
	CK49	10	320 (3 months)
Group 4- (1.4e14 GC/kg)	CP26	20	5120 (6 months)
	BM79	80	2560 (6 months)

Table S5. External observations and gross necropsy results

BSC, body composition score; GC/kg, genome copies per kilogram; SIV, simian immunodeficiency virus.

	Animal ID	Date of necropsy	Final weight (kg)	Body weight change from pre-infection (%)	External observations	Gross necropsy results
Group 1 untreated 3 months	1T6	2/04/19	3.2	-10.3	thin; 7% to 10% dehydrated, body condition score 2/5	dehydrated, thin body condition (2/5 BCS), findings consistent with SIV infection
	7S0	2/04/19	4.7	+2.1	subcutaneous abscess at ART injection sites	no significant findings
	CF53	2/11/19	3.9	+8.1	no significant findings	no abnormal or significant findings
Group 2 1.4 x 10^{12} GC/kg, 3 months	CF63	2/11/19	4.1	+7.5	no significant findings	no abnormal or significant findings
	CI77	2/12/19	5.2	+23.7	no significant findings	no abnormal or significant findings
	MA285	2/12/19	5.1	+13.6	no significant findings	no abnormal or significant findings
Group 3 1.4 x 10^{13} GC/kg, 3 months	CK10	2/05/19	4.2	-12.8	no significant findings; weight loss of 10% from highest weight but body condition score was in normal range 3/5	no abnormal or significant findings (weight loss noted)
	CK49	2/05/19	5.2	+14.3	no significant findings	no abnormal or significant findings
Group 3 1.4 x 10^{13} GC/kg, 6 months	CF27	4/15/19	6.0	+11.3	no significant findings	no abnormal or significant findings
	CH97	4/15/19	4.7	+7.1	no significant findings	no abnormal or significant findings
Group 4 1.4 x 10^{14} GC/kg, 6 months	BM79	8/10/20	4.9	+51.2	no significant findings	no abnormal or significant findings
	CP26	8/11/20	4.2	+25.4	no significant findings	no abnormal or significant findings

Table S6. Excision (5G and G3) by EBT-001 in SIV-infected antiretroviral treated non-human primates at necropsy

All group 1 samples were negative for excision in both assays. No virus was detected in mesenteric lymph node, brain frontal of 7S0, bone marrow of CF53 and IT6 and heart left atrium of IT6. 5G, 5G excision assay; G3, G3 excision assay; -, no excision product was detected for that tissue for any replicates of the specific pcr assay; +, positive excision as determined by the correct size amplicon and sequence in at least one of the replicates; NV, no specific viral product (full-length or excision product) was amplified for the 5G excision assay; SIV, simian immunodeficiency virus

		Group 2			Group 3 (3 months)		Group 3 (6 months)		Group 4	
Animal Number		CF63	CI77	MA285	CK10	CK49	CF27	CH97	BM79	CP26
Spleen	5G	-	-	+	+	-	-	-	-	-
	G3	-	-	+	+	-	+	-	-	-
Mesenteric lymph node	5G	+	+	+	-	+	-	-	+	-
	G3	-	+	+	-	+	+	+	-	-
Liver	5G	+	+	+	+	+	+	-	+	NV
	G3	+	+	+	+	+	+	-	-	-
Kidney	5G	+	-	+	+	+	-	NV	+	NV
	G3	-	+	+	-	+	+	+	-	-
Heart- right ventricle	5G	+	-	+	-	-	+	NV	NV	-
	G3	+	-	+	-	-	-	+	-	+
Heart- left ventricle	5G	+	+	+	+	+	+	-	-	NV
	G3	+	+	+	+	+	+	-	-	-
Heart- right atrium	5G	+	+	+	+	-	+	-	NV	-
	G3	+	-	+	+	+	+	+	+	+
Heart- left atrium	5G	+	+	+	+	+	-	+	NV	-
	G3	+	+	-	+	+	+	+	-	+
Testes	5G	+	-	+	-	+	+	NV	+	-
	G3	+	-	+	-	+	+	-	-	+
Lung	5G	+	+	-	+	+	+	-	+	-
	G3	+	+	+	+	+	+	+	+	+
Brain- prefrontal	5G	+	+	-	+	+	+	-	NV	NV
	G3	+	+	+	+	+	+	-	+	+
Brain- frontal	5G	+	+	+	+	+	+	-	NV	NV
	G3	+	+	+	+	+	+	+	-	-
Brain- parietal	5G	+	+	+	+	+	+	+	NV	-

	G3	+	+	+	+	+	+	+	-	+
Brain- temporal	5G	+	+	+	+	+	-	NV	+	NV
	G3	+	-	+	+	-	+	+	-	-
Brain- occipital	5G	+	+	+	+	+	+	NV	NV	-
	G3	-	+	+	+	+	+	+	-	-
Brain- cerebellum	5G	+	+	+	+	+	+	+	NV	+
	G3	+	+	+	+	-	+	+	-	+
Brain- brain stem	5G	+	+	+	+	+	+	NV	NV	-
	G3	+	+	+	+	+	+	+	+	+
Bone marrow	5G	+	+	+	+	+	-	NV	+	-
	G3	+	+	+	+	+	+	-	+	-
Colon	5G	-	-	-	-	-	-	-	-	-
	G3	-	-	+	-	-	+	-	-	-
Duodenum	5G	-	-	+	-	+	+	-	-	-
	G3	-	-	+	-	-	-	-	-	-

Table S7. Longitudinal examination of excision of blood by EBT-001 in SIV-infected antiretroviral treated non-human primates

All group 1 samples were negative for excision in both assays. 5G, 5G excision assay; G3, G3 excision assay; - no excision product was detected for that tissue for the specific pcr assay; +, positive excision as determined by the correct size amplicon and sequence in at least one of the replicates; ND, not done, no blood due to the COVID lockdown; N/A, not applicable as these animals were necropsied at 3-months post-EBT-001; SIV, simian immunodeficiency virus.

		Group 2			Group 3 (3 months)		Group 3 (6 months)		Group 4	
Blood		CI77	CF63	MA285	CK49	CK10	CF27	CH97	BM79	CP26
2 weeks	5G	+	-	-	-	+	-	-	ND	ND
	G3	-	-	-	-	-	-	-	ND	ND
6 weeks	5G	-	-	-	-	+	-	-	ND	ND
	G3	-	-	+	-	+	-	-	ND	ND
10 weeks	5G	+	-	+	-	+	+	+	ND	ND
	G3	+	+	+	-	+	+	+	ND	ND
4 months	5G	N/A	N/A	N/A	N/A	N/A	-	-	-	-
	G3	N/A	N/A	N/A	N/A	N/A	-	+	+	+
5 months	5G	N/A	N/A	N/A	N/A	N/A	-	-	+	-
	G3	N/A	N/A	N/A	N/A	N/A	-	-	+	+
Necropsy	5G	-	-	-	-	-	-	-	+	-
	G3	-	-	-	-	-	-	-	-	-

]Table S8. Percent of 5G excision in tissues of EBT-001-treated rhesus macaques.

NV, no full-length virus, or excised amplicon seen in the 5G EXA.

5G-Efficiency %	Group 2			Group 3 (3 months)		Group 3 (6 months)		Group 4	
Animal number	CF63	CI77	MA285	CK10	CK49	CF27	CH97	BM79	CP26
Spleen	0	0	100	9	0	0	0	0	0
Mesenteric lymph node	40	61	100	0	35	0	0	100	0
Liver	100	100	100	100	100	100	0	100	NV
Kidney	100	0	100	7	68	0	NV	100	NV
Heart- right ventricle	100	0	100	0	0	100	NV	NV	0
Heart- left ventricle	63	100	100	100	100	100	0	0	NV
Heart- right atrium	100	100	63	78	0	74	0	NV	0
Heart- left atrium	100	100	100	100	57	0	100	NV	0
Testes	100	0	100	0	59	100	NV	9	0
Lung	100	100	0	100	100	15	0	100	0
Brain- prefrontal	100	100	0	100	100	100	0	NV	NV
Brain- frontal	100	100	100	100	100	100	NV	NV	NV
Brain- parietal	100	100	100	100	100	100	100	NV	0
Brain- temporal	100	100	100	100	100	0	NV	100	NV
Brain- occipital	100	100	100	100	100	100	NV	NV	0
Brain- cerebellum	100	100	100	100	100	100	100	NV	26
Brain- brain stem	100	100	100	100	100	76	NV	NV	0
Bone marrow	100	100	100	100	100	0	NV	100	0
Colon	0	0	0	0	0	0	0	0	0
Duodenum	0	0	36	0	34	74	0	0	0

Table S9. Percent of 5G excision in serial blood samples of EBT-001-treated rhesus macaques

All group 1 samples were negative for excision in this assay. ND, not done, no blood due to the COVID lockdown; N/A, not applicable as these animals were necropsied at 3-months post-EBT-001.

5G Efficiency %	Group 2			Group 3 (3 months)		Group 3 (6 months)		Group 4	
Months post-EBT-001	CI77	CF63	MA285	CK49	CK10	CF27	CH97	BM79	CP26
0.5	31	0	0	0	44	0	0	ND	ND
1.5	0	0	0	0	73	0	0	ND	ND
2.5	80	0	100	0	70	100	53	ND	ND
4	N/A	N/A	N/A	N/A	N/A	0	0	0	0
5	N/A	N/A	N/A	N/A	N/A	0	0	41	0
Necropsy (3 or 6)	0	0	0	0	0	0	0	100	0

Table S10. Liver function tests in NHP following EBT-001 at a dose of 1.4 x 10^{14} GC/kg

[a]Baseline value is the last pre-dose value (Day 0). The range of pre-dose values is noted in parentheses. [b]The first post-dose laboratory evaluations were obtained on Day 6. Shown are peak values that all occurred on Day 6 with exception of ALP for BM79, which occurred on Day 55. [c]Day post dosing when value returned to BL range. ALP: alkaline phosphatase; ALT: alanine aminotransferase; AST: aspartate aminotransferase; BL: baseline; NHP: non-human primate.

Parameter		Animal number	
		CP26	BM79
ALP (U/L)	Baseline Value (Range) [a]	449 (289-449)	473 (219-445)
	Peak Value [b]	840	542
	Return to BL - Value	429	441
	Return to BL - Day [c]	48	62
ALT (U/L)	Baseline (Range)	30 (24-125)	28 (24-80)
	Peak Value	1530	351
	Return to BL - Value	89	51
	Return to BL - Day	27	19
AST (U/L)	Baseline (Range)	27 (27-92)	33 (21-98)
	Peak Value	933	170
	Return to BL - Value	88	50
	Return to BL - Day	19	13
Total bilirubin (mg/dL)	Baseline (Range)	0.1 (0.1-0.2)	0.2 (0.1-0.2)
	Peak Value	6.5	0.8
	Return to BL - Value	0.2	0.2
	Return to BL - Day	34	19

Table S11. Inspection of genomic sites identified with SNP changes. Representative supporting reads were shown for each site listed in Table 2. Further inspection was performed by inspecting the reads with its corresponding aligned chromosomal region. *Bolded text: hypothetical cut sites. Red highlight: differences in the read compared to the chromosomal sequence.

<table>
<tr><th>Nominated Site ID</th><th>Monkey</th><th>Ref</th><th>SNP</th><th>Distance from hypothetical cut site</th><th>Sequence alignment of chromosome sequences (top) against representative reads (bottom) with match strings (middle) *</th><th>Notes</th></tr>
<tr><td>SIV_031_6MM_gag_03</td><td>CH97</td><td>G</td><td>+T</td><td>7</td><td rowspan="2">TTTAACCATTTTAAAGTACATAGTTCGGTGGCATTAAGTG
||||||||||||||||||||||||||||||||||||||||
TTTAACCATTTTAAAGTACATAGTTCGGTGGCATTAAGTG

aATTCACGTTG-AGTGCGGCCATCGCCACCATCCATCTCC
 ||||||||||-||||||||||||||||||||||||||||
cATTCACGTTGtAGTGCGGCCATCGCCACCATCCATCTCC

AGAATGTTTTCGTCATCCTGAA
||||||||||||||||||||||
AGAATGTTTTCGTCATCCTGAA</td><td rowspan="2">SNP is heterozygous at baseline, and locates in an intron of LARGE xylosyl- and glucuronyltransferase 1. Unlikely to have biological impact even if this was a result of editing.</td></tr>
<tr><td>SIV_031_6MM_gag_03</td><td>CK49</td><td>G</td><td>+T</td><td>7</td></tr>
<tr><td>SIV_052_4MM_ltr_13</td><td>CH97</td><td>T</td><td>+A</td><td>−10</td><td rowspan="2">GTACCCAGCAAGTGCTCAGGGCTCACGTTACAGAGGGGAAGTC
|||||||||||||||||||||||||||||||||||||||||||
GTACCCAGCAAGTGCTCAGGGCTCACGTTACAGAGGGGAAGTC

AGGCAGAT-AGTGAGGTACCCAGCAGGGTTCACGTTACCAAGG
||||||||-||||||||||||||||||||||||||||||||||
AGGCAGATaAGTGAGGTACCCAGCAGGGTTCACGTTACCAAGG

AGGAAAGGAGCGTGGA
||||||||||||||||
AGGAAAGGAGCGTGGA</td><td rowspan="2">Indel occurred at a distant site from the hypothetical cut site.</td></tr>
<tr><td>SIV_052_4MM_ltr_13</td><td>CK49</td><td>T</td><td>+A</td><td>−10</td></tr>
<tr><td>SIV_093_5MM_ltr_44</td><td>CK49</td><td>A</td><td>+T</td><td>−10</td><td>CACCTTTCTGGTAGCTGGGACTGCAGGCATGTGCCACCATAC
||||||||||||||||||||||||||||||||||||||||||</td><td></td></tr>
</table>

<table>
<tr><td>SIV_093_5MM_ltr_44</td><td>CH97</td><td>A</td><td>+T</td><td>−10</td><td>CACCTTTCTGGTAGCTGGGACTGCAGGCATGTGCCACCATAC

CCAGCTGAA-TTTTTTTTTTTTCATACAGTGGAATTTAATTC
|||||||||-||||||||||||||||||||||||||||||||
CCAGCTGAAtTTTTTTTTTTTTCATACAGTGGAATTTAATTC

AGTGATACTCAGTTATAA
||||||||||||||||||
AGTGATACTCAGTTATAA</td><td>The insertion is likely due to sequencing error caused by long stretch of Ts.</td></tr>
<tr><td>SIV_130_5MM_ltr_73</td><td>CK49</td><td>G</td><td>-</td><td>7</td><td>TCAGCCTCCCGAGTAGCTGGGATTgCAGGCATGTGtCACCACA
|||||||||||||||||||||||| |||||||||| ||||||
TCAGCCTCCCGAGTAGCTGGGATTaCAGGCATGTGcCACCACA

CCCAGaTgAgTTTTGtATTTTTAGTAGAGATGGGaTTTCACCA
|||||.| | ||||| |||||||||||||||||| ||||||||
CCCAG-TtAaTTTTGcATTTTTAGTAGAGATGGGgTTTCACCA

TGTTGGtCAGGCTGc
|||||| |||||||
TGTTGGcCAGGCTGg</td><td rowspan="2">Two representative supporting reads both contain many differences compared to the chromosome location, which is likely due to poor sequencing and/or alignment.</td></tr>
<tr><td>SIV_130_5MM_ltr_73</td><td>CH97</td><td>G</td><td>-</td><td>7</td><td>TCAGCCTCCCGAGTAGCTGGGATTgCAGGCATGTGtCACCACA
|||||||||||||||||||||||| |||||||||| |||||||
TCAGCCTCCCGAGTAGCTGGGATTaCAGGCATGTGcCACCACA

CCCAGaTgAgTTTTGTATTTTTAGTAGAGA
||||| |.| ||||||||||||||||||||
CCCAGcT-AaTTTTGTATTTTTAGTAGAGA</td></tr>
<tr><td>SIV_135_4MM_gag_03</td><td>CK49</td><td>A</td><td>+C</td><td>10</td><td>CTTTCTATTACaCAGTTTTGAAACTGCGTCATtGAtGGATTCA
||||||||||| |||||||||||||||||||| || |||||||</td><td></td></tr>
</table>

<table>
<tr><td>SIV_135_
4MM_gag
_03</td><td>CH97</td><td>A</td><td>+C</td><td>10</td><td>CTTTCTATTACtCAGTTTTGAAACTGCGTCATgGAgGGATTCA

CGAaGGGA-ATTTcGGGAGCACATCTTTCTATTACACAGTTTT
||| ||||-||||.|||||||||||||||||||||||||||||
CGAcGGGAcATTT-GGGAGCACATCTTTCTATTACACAGTTTT

GAAACTGCgTCATtGAtgGATTCACGAAGGGA-ATTTcGGGAG
|||||||| |||| || ||||||||||||||-||||.|||||
GAAACTGCcTCATgGAcaGATTCACGAAGGGAcATTT-GGGAG

cACAtaCAGgtcTgTGGGGAAATAGGAAATATCT
 ||| ||| | ||||||||||||||||||||
tACActCAGacaTcTGGGGAAATAGGAAATATCT</td><td>The supporting reads contain many differences compared to the chromosome location, which is likely due to poor sequencing and/or alignment.</td></tr>
</table>

FIGURES

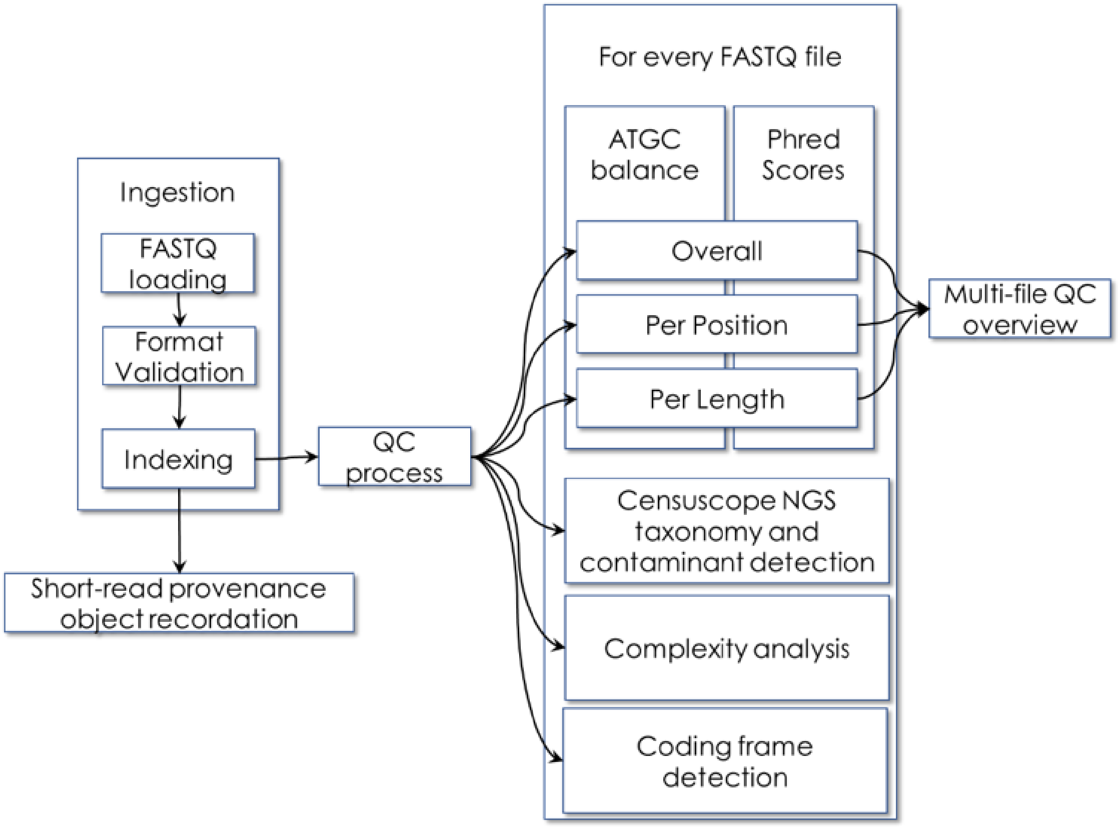

Figure S1. **Data ingestion and quality control procedures.** NGS, next generation sequencing; QC, quality control

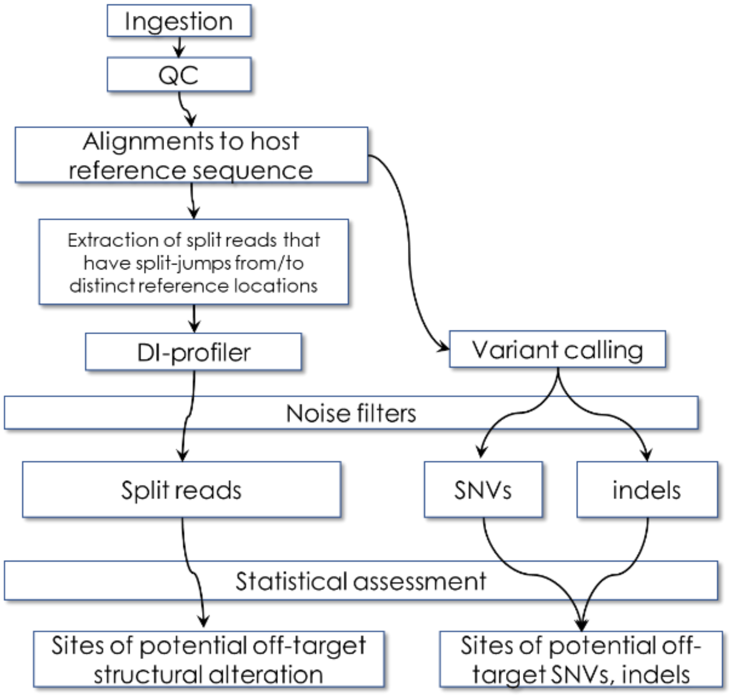

Figure S2. **Pipeline for the discovery of off-target SNVs, indels, structural alterations, and AAV integration.**

Indels, insertions and deletions; QC, quality control; SNV, single nucleotide variant.

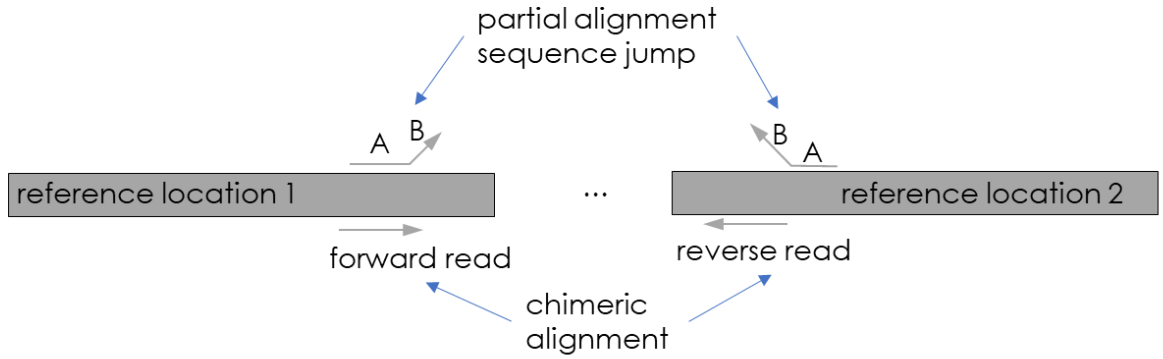

Figure S3. **Schematic representation of partial and chimeric alignments for different types of split read sequence jumps**

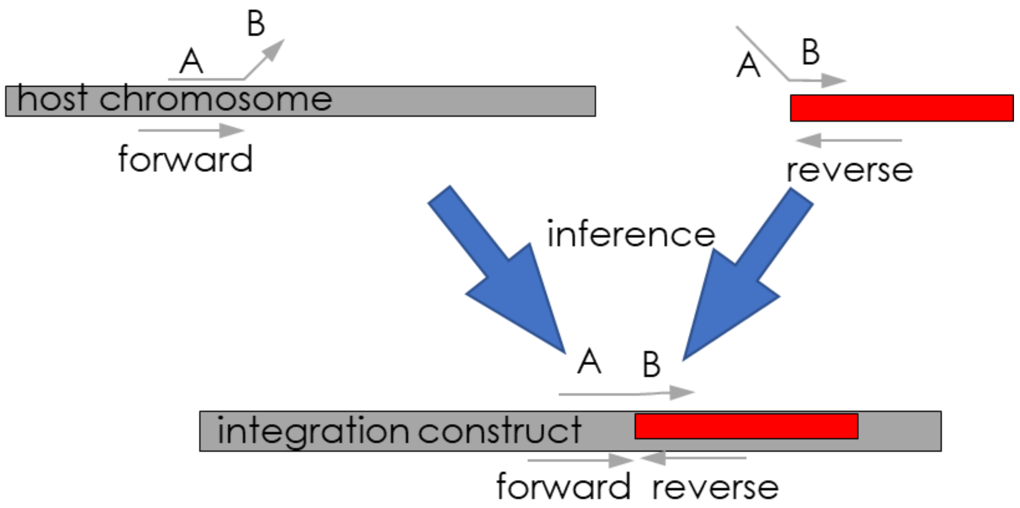

Figure S4. **Schematic representation of partial and chimeric alignment inference of a viral integration site**

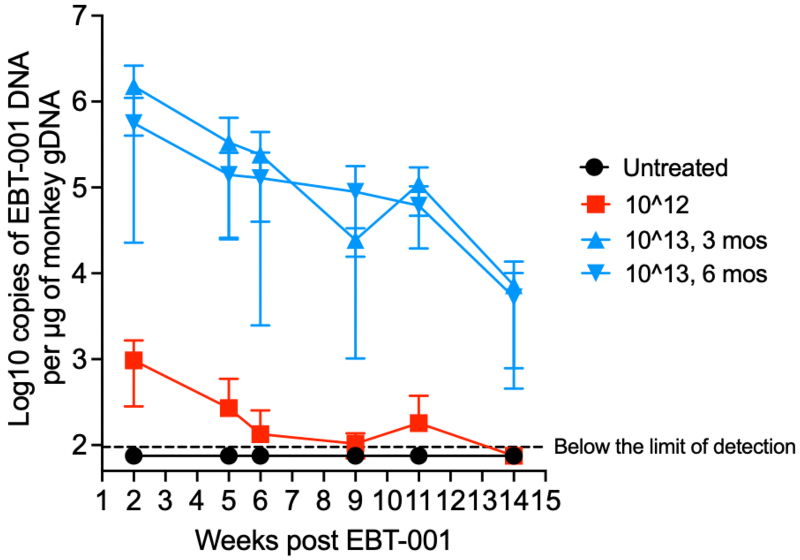

Figure S5. Biodistribution in blood weeks after EBT-001.

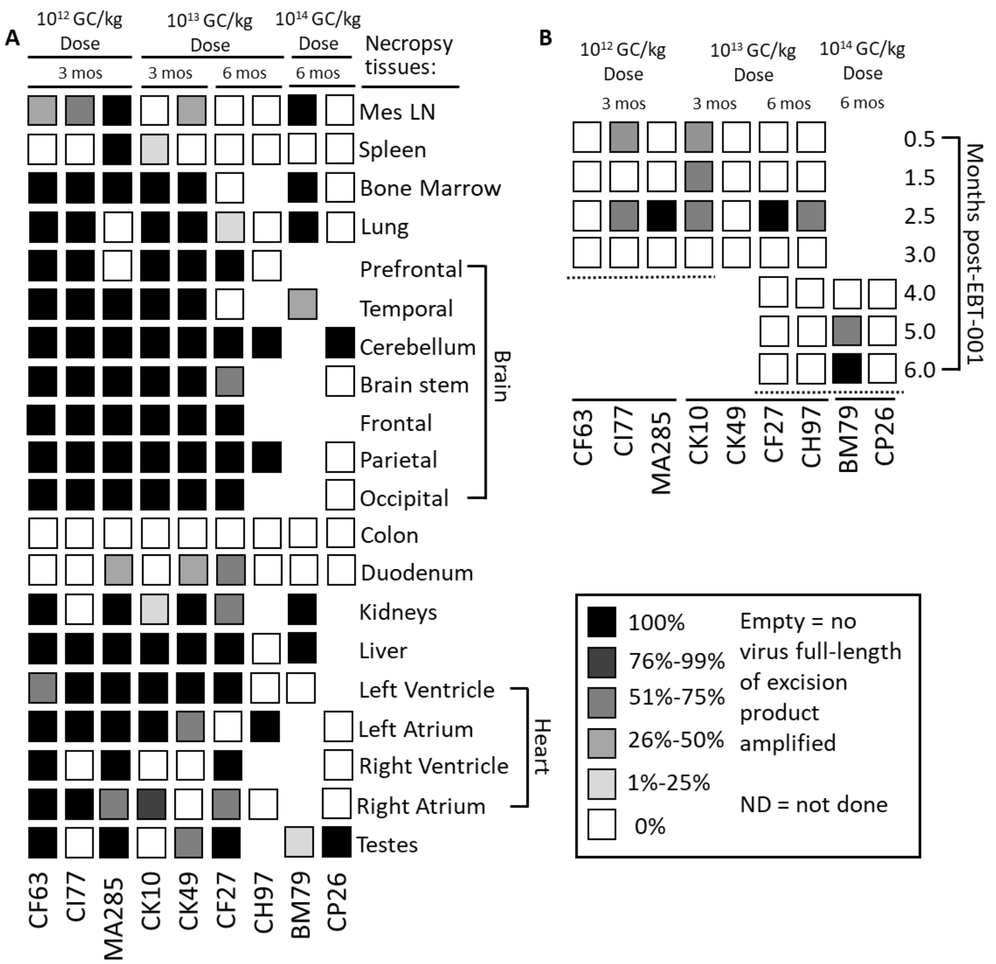

Figure S6. Heat map of 5G viral excision efficiency demonstrates activity across tissues

A. The percent of viral excision in the 5G EXA assay in tissues at necropsy and **B.** in blood after EBT-001. Empty spaces mean there was no full-length or excised virus was detected in the 5G EXA. Black boxes represent 100% excision efficiency (no full-length and only excised band) and white boxes represent 0% excision, which is no excision band but only full-length present. The light to dark gray boxes are increasing percentages of excision as described in the legend. ND, not done, no blood due to the COVID lockdown.

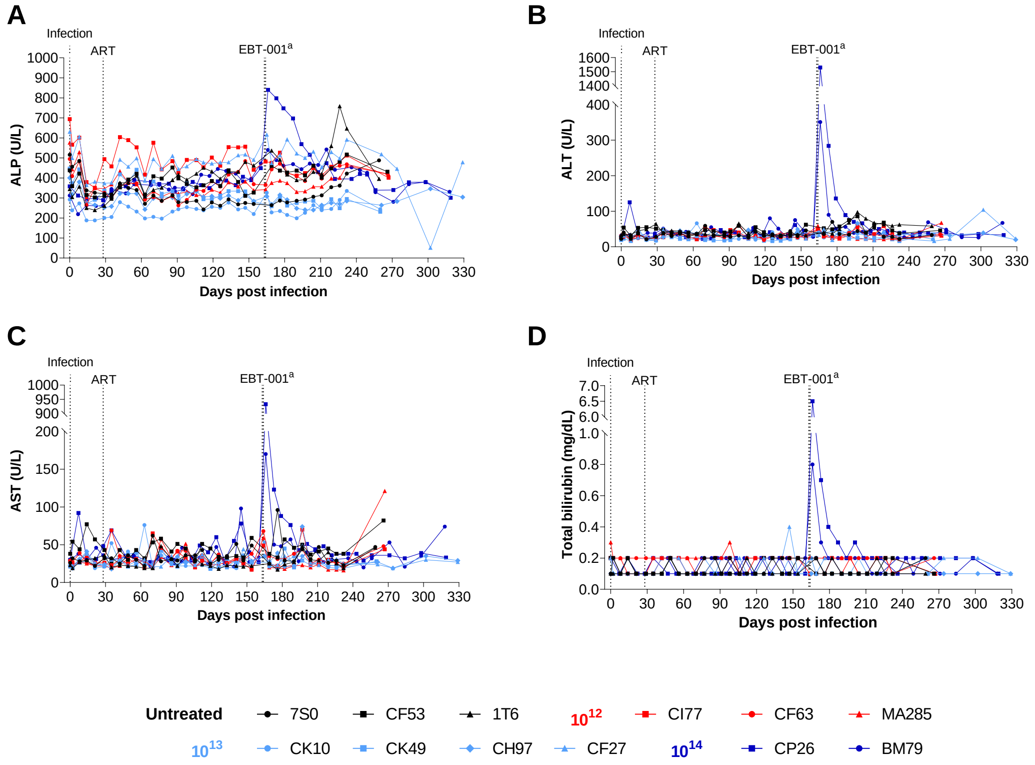

Figure S7. Assessment of liver function over time. Animals were SIV infected at Day 0 with monitoring beginning up to 6 days prior. ART was administered 28 days after infection. EBT-001 was administered ~160-170 days after infection. alkaline phosphatase (ALP) (**A**), alanine transaminase (ALT) (**B**), aspartate aminotransferase (AST) (**C**), and in total serum bilirubin (**D**) were assessed over time in EBT-001-treated (red and blue) and untreated animals (black). [a]CK49 received ~1/2 of EBT-001 split across 2 days, 4 days apart. ALP, alkaline phosphatase; ALT, alanine transaminase; ART, antiretroviral therapy; AST, aspartate transaminase.

<table>
<tr><th></th><th>Sequence</th><th>Chromosome</th><th>Start</th><th>End</th></tr>
<tr><td>A</td><td>TCCGTTTGCGTAGTGTTTCTCCAGCTCTTTACTTTAAGCCTGTTGGTGTAGCATGAAATGGGTCTCTTaAAGATAACAGA</td><td>Chr7</td><td>128862755</td><td>128862834</td></tr>
<tr><td></td><td>|||||||||||||||||||||||||||||||||||||||||||||||||||||||||||||||||||| |||||||||||</td><td>Match string</td><td></td><td></td></tr>
<tr><td></td><td>TCCGTTTGCGTAGTGTTTCTCCAGCTCTTTACTTTAAGCCTGTTGGTGTAGCATGAAATGGGTCTCTTgAAGATAACAGA</td><td>Read1</td><td>81</td><td>2</td></tr>
<tr><td></td><td></td><td></td><td></td><td></td></tr>
<tr><td></td><td>CGGACCCCATTCCAGTACCTGAGCAGCAGCGACAGCAAGATCAGCTACGAAACCTTCAAGAAGCACATCCTG</td><td>EBT001</td><td>2565</td><td>2636</td></tr>
<tr><td></td><td>||||||||||||||||||||||||||||||||||||||||||||||||||||||||||||||||||||||||</td><td>Match string</td><td></td><td></td></tr>
<tr><td></td><td>CGGACCCCATTCCAGTACCTGAGCAGCAGCGACAGCAAGATCAGCTACGAAACCTTCAAGAAGCACATCCTG</td><td>Read1</td><td>78</td><td>149</td></tr>
<tr><td>B</td><td>GGAGTCTCACTCTGTTGTCCAGGCTGGAGTGCAGTGGCGCAATC</td><td>Chr12</td><td>28738543</td><td>28738586</td></tr>
<tr><td></td><td>||||||||||||||||||||||||||||||||||||||||||||</td><td>Match string</td><td></td><td></td></tr>
<tr><td></td><td>GGAGTCTCACTCTGTTGTCCAGGCTGGAGTGCAGTGGCGCAATC</td><td>Read2</td><td>106</td><td>149</td></tr>
<tr><td></td><td></td><td></td><td></td><td></td></tr>
<tr><td></td><td>ATTTCATCTTCAAAGAGTGGAAGAAACTGGACAAGGCCAAAAAAGTGATGGAAAACCAGATGTTCGAGGAAAAGCAGGCCGAGAGCATGCCCGA</td><td>EBT001</td><td>2944</td><td>3037</td></tr>
<tr><td></td><td>||||||||||||||||||||||||||||||||||||||||||||||||||||||||||||||||||||||||||||||||||||||||||||||</td><td>Match string</td><td></td><td></td></tr>
<tr><td></td><td>ATTTCATCTTCAAAGAGTGGAAGAAACTGGACAAGGCCAAAAAAGTGATGGAAAACCAGATGTTCGAGGAAAAGCAGGCCGAGAGCATGCCCGA</td><td>Read2</td><td>95</td><td>2</td></tr>
</table>

Figure S8. Split reads with partial Cas9 homology. A) 1 representative read from CH97 post treatment with partial alignment to Chr7:128862755-128862834 with partial alignment to Cas9 coding region (EBT001: 2636-2565). B) 1 read in CK49 post treatment with partial alignment to Cas9 coding region (EBT001: 2944-3037), and partially aligned to Chr12:28738543-28738586.
